# Multiplexed capture of spatial configuration and temporal dynamics of locus-specific 3D chromatin by biotinylated dCas9

**DOI:** 10.1186/s13059-020-01973-w

**Published:** 2020-03-05

**Authors:** Xin Liu, Yong Chen, Yuannyu Zhang, Yuxuan Liu, Nan Liu, Giovanni A. Botten, Hui Cao, Stuart H. Orkin, Michael Q. Zhang, Jian Xu

**Affiliations:** 1 Children’s Medical Center Research Institute, University of Texas Southwestern Medical Center, Dallas, TX 75390 USA; 2 Department of Pediatrics, Harold C. Simmons Comprehensive Cancer Center, and Hamon Center for Regenerative Science and Medicine, University of Texas Southwestern Medical Center, Dallas, TX 75390 USA; 3 Department of Biological Sciences, Center for Systems Biology, University of Texas at Dallas, Richardson, TX 75080 USA; 4 Department of Molecular and Cellular Biosciences, Rowan University, Glassboro, NJ 08028 USA; 5 Division of Hematology/Oncology, Boston Children’s Hospital, Dana-Farber Cancer Institute and Harvard Stem Cell Institute, Harvard Medical School, Boston, MA 02115 USA; 6 Howard Hughes Medical Institute, Boston, MA 02115 USA

**Keywords:** CRISPR/Cas9, Chromatin, Epigenetics, 3D genome, Enhancers

## Abstract

The spatiotemporal control of 3D genome is fundamental for gene regulation, yet it remains challenging to profile high-resolution chromatin structure at *cis*-regulatory elements (CREs). Using C-terminally biotinylated dCas9, endogenous biotin ligases, and pooled sgRNAs, we describe the dCas9-based CAPTURE method for multiplexed analysis of locus-specific chromatin interactions. The redesigned system allows for quantitative analysis of the spatial configuration of a few to hundreds of enhancers or promoters in a single experiment, enabling comparisons across CREs within and between gene clusters. Multiplexed analyses of the spatiotemporal configuration of erythroid super-enhancers and promoter-centric interactions reveal organizational principles of genome structure and function.

## Background

The eukaryotic genome is hierarchically organized into multiscale domains by 3D chromatin interactions [1–3]. The spatial and temporal regulation of higher-order chromatin organization modulates gene transcription that in turn controls cellular phenotypes in development and disease [4–8]. Recent genome-scale analysis of the 3D genome uncovered multilayered structural units including compartments, topologically associating domains (TADs), and chromatin loops [9–14]. At the highest resolution, chromatin loops mediate enhancer-promoter interactions to control tissue- and developmental stage-specific gene expression [13, 14]. Currently, major challenges are to elucidate the underlying principles of chromatin organization and to understand how chromatin interactions between *cis*-regulatory elements (CREs) relate to gene activity [15, 16].

Current technologies in studying 3D genome rely on chromatin conformation capture (3C)-based nuclear proximity ligation to detect interacting DNA fragments tethered together by long-range chromatin loops [15]. While Hi-C [9] and ChIA-PET [17] technologies have enabled systematic interrogations of genome-scale landscapes of chromatin interactions, they often lack the level of resolution required to evaluate the spatial organization of locus-specific interactions including enhancer-promoter looping, as well as their temporal dynamics during cellular differentiation [1, 16]. They also do not provide information about the composition of *trans*-acting factors that regulate long-range chromatin interactions. To isolate endogenous CRE-regulating chromatin complexes and 3D structure, we previously developed the dCas9-based CAPTURE (CRISPR Affinity Purification in situ of Regulatory Elements) method [18–20]. By co-expression of the biotin-acceptor-site-containing dCas9, bacterial BirA biotin ligase, and target-specific sgRNAs, the locus-bound dCas9 complexes are in vivo biotinylated and isolated by streptavidin-based affinity purification, followed by analyses of locus-associated protein factors and long-range DNA interactions using proteomics and 3C-based methodologies, respectively [18–20]. The CAPTURE method provides a complementary method for unbiased analysis of locus-specific chromatin interactions without predefined protein factors or a priori knowledge of the target loci.

The original CAPTURE protocol [18, 19] relies on a single or several sgRNAs to isolate a single CRE in mammalian genomes, which requires large numbers of cells for proteomics (10^8^~10^9^) or 3C-based (5 × 10^7^) analyses. Because of the cell number requirements, the experimental designs involve the generation of stable cell lines co-expressing CAPTURE components, thus are not applicable to primary tissues or rare cell populations, suggesting that the capture sensitivity and efficiency need to be improved for low cell numbers. Moreover, the single locus-based capture requires independent experiments by individual sgRNAs targeting discrete CREs and is subject to variations in experimental conditions (e.g., dCas9 and sgRNA expression levels in different cell lines). Thus, it does not allow comparisons between different CREs in a single experiment or the same CREs at different stages of cellular differentiation [18].

Hence, there remains a need for the new methodology that can map the spatial organization and temporal dynamics of chromatin structure of many genomic loci at once in living cells. The method should be applicable to any genomic loci and capture full interaction profiles at high resolution, enabling systematic comparisons across CREs within and between gene clusters or developmental stages. Here, we describe the significantly redesigned CAPTURE2.0 method for multiplexed, high-throughput, and high-resolution analysis of CRE-mediated 3D chromatin structure. The new system enables quantitative analysis of the spatial configuration of a few to hundreds of enhancers or promoters in a single experiment. Multiplexed analyses of erythroid super-enhancers (SEs) reveal SE hierarchical structure and distinct modes of SE-gene interactions. High-throughput capture of promoter-centric interactions establishes the instructive function of developmentally controlled enhancer-promoter loops in transcriptional regulation and lineage differentiation. These applications illustrate the ability of multiplexed CAPTURE for decoding the organizational principles of genome structure and function.

## Results

### A redesigned CAPTURE system by C-terminal biotin-labeled dCas9

To isolate locus-specific chromatin interactions, we previously developed the CAPTURE method by co-expressing the N-terminal FLAG-biotin (FB)-tagged dCas9, BirA biotin ligase, and sgRNAs (Additional file 1: Figure S1a; CAPTURE1.0). Upon in vivo biotinylation, the locus-bound dCas9 was isolated by streptavidin-based affinity purification, followed by the analysis of locus-associated protein factors and long-range DNA interactions [18–20]. The original CAPTURE1.0 system required the generation of stable cell lines co-expressing three components (FB-dCas9, BirA, and sgRNA), thus was not applicable to primary cells or rare cell populations. Moreover, the single locus-based capture did not allow comparisons between different genomic loci in a single experiment [18, 19].

Several critical factors influence the efficiency of CAPTURE assays. First, the efficacy of the sequence-specific sgRNA in directing dCas9 to its target genomic locus is important and is largely dictated by the underlying sequences [21, 22]. Second, the expression levels of dCas9 and sgRNA need to be optimized to maximize dCas9 on-target binding and minimize non-specific signals [18, 19]. Third, the efficiency of in vivo biotinylation of dCas9 protein is critical for the purification of dCas9-tethered genomic loci from the cellular milieu. To develop a widely applicable technology for analyzing locus-specific chromatin structures across cell types, we sought to evolve the CAPTURE method by multiple iterations considering factors that influence capture efficiency. We first engineered a tricistronic vector containing BirA, FB-dCas9, and zsGreen1 to allow for co-expression of BirA and dCas9 from a single transcript (Additional file 1: Figure S1a; CAPTURE1.1). We next replaced the FLAG-biotin-tag with a BioTAP-tag [23, 24], which contains a 69-amino-acid biotinylation targeting sequence that is recognized by endogenously expressed biotin protein ligases (BPL) in eukaryotic cells [23, 25]. More importantly, we noted that the N-terminus of dCas9 is in close proximity to target DNA recognition domain that lies within the PAM-recognition cleft [26, 27], which might result in epitope masking that interferes with in vivo biotinylation. In contrast, the C-terminus of dCas9 is largely unstructured and exposed [26, 27]. Therefore, we engineered two versions of CAPTURE2.0 using N- and C-terminal biotin-tagged dCas9 (Additional file 1: Figure S1a; CAPTURE2.0 NBio and CBio).

We then compared the capture efficiency and on-target enrichment of different CAPTURE systems by CAPTURE-ChIP-qPCR analysis of the human β-globin gene promoters (*HBG1*, *HBG2*, and *HBB*) in K562 cells. By co-expressing CAPTURE1.0 (FB-dCas9 and BirA) and a validated sgRNA targeting *HBG1/2* promoters [18], we detected 740-fold on-target enrichment relative to the nearby non-targeted *HBB* promoter (Additional file 1: Figure S1b). The bicistronic CAPTURE1.1 system modestly improved the capture efficiency by a 4.8-fold increase in ChIP signal at *HBG1/2* promoters (8.4% vs 1.7% of input DNA) and increased on-target enrichment (2668-fold). Importantly, both NBio- and CBio-CAPTURE2.0 systems markedly increased the capture efficiency (Additional file 1: Figure S1b). The C-terminal biotinylation of dCas9 resulted in a 13.6-fold increase in capture efficiency relative to CAPTURE1.0 (23.7% vs 1.7% of input DNA), whereas the on-target enrichment was largely comparable between different CAPTURE systems. These results demonstrate that the redesigned CAPTURE2.0 system by C-terminal biotin-tagging significantly improved the capture efficiency while retaining high specificity and on-target enrichment for purification of dCas9-targeted chromatin.

### Multiplexed CAPTURE of β-globin locus control region

**Fig. 1:**
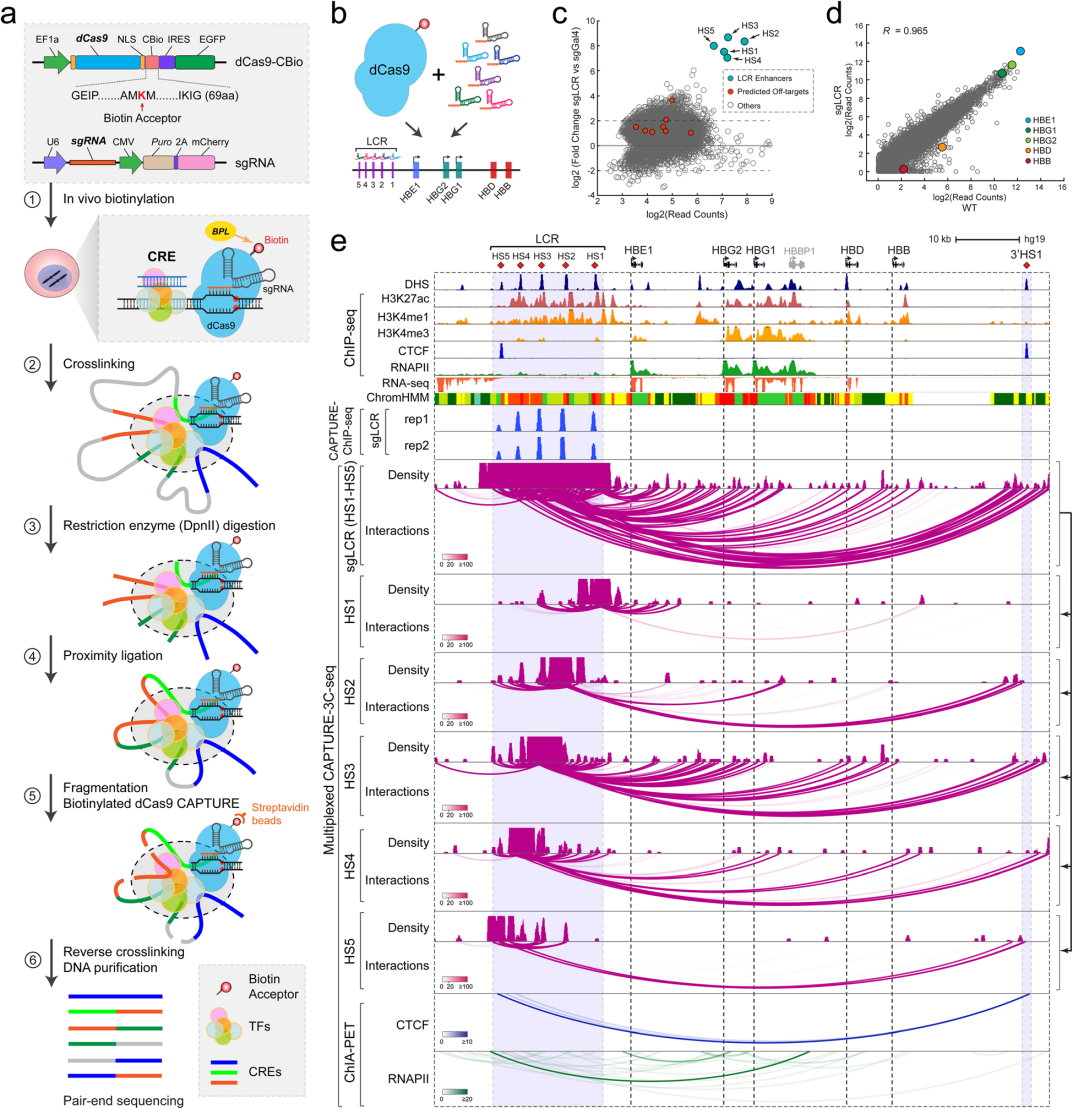
Multiplexed CAPTURE of locus-specific long-range DNA interactions. **a** Schematic of multiplexed analysis of locus-specific chromatin interactions by the redesigned CAPTURE2.0 system containing the C-terminal biotin-tagged dCas9 (dCas9-CBio) and sgRNA. Major steps of the CAPTURE-3C-seq method are shown. **b** Schematic of dCas9-mediated multiplexed capture of the human β-globin LCR. **c** Genome-wide analysis of dCas9 binding in cells expressing LCR-targeting sgRNAs (sgLCR) or non-targeting sgGal4. Data points for the sgRNA target regions are shown by arrowheads, and the predicted off-targets are shown as red dots. The *x*- and *y*-axes denote the log2 mean read counts and the log2 ratio of read counts in sgLCR and sgGal4 samples from *N* = 2 and 4 CAPTURE-ChIP-seq experiments, respectively. **d** Genome-wide differential gene expression analysis was performed using RNA-seq in K562 cells expressing dCas9-CBio with sgLCR or wild-type (WT) K562 cells. The β-like globin genes are indicated by colored data points. Pearson correlation coefficient (*R*) value is shown (*N* = 2 RNA-seq experiments). **e** Browser view of LCR-mediated long-range interactions (chr11: 5,222,424-5,323,623; hg19) is shown. Contact profiles including the density map and interactions (or loops) for the dCas9-captured LCR or the resolved individual HS regions are shown. The statistical significance of interactions was determined by the Bayes factor (BF) and indicated by the color scale bars. DHS, ChIP-seq, RNA-seq, ChromHMM, CAPTURE-ChIP-seq (sgLCR), and ChIA-PET (CTCF and RNAPII) data are shown for comparison

To validate the redesigned CAPTURE system for characterizing locus-specific chromatin interactions, we focused on the CAPTURE-3C-seq method [18]. Specifically, we combined high-affinity dCas9 capture with 3C-based chromatin interaction assays [15] to identify locus-specific long-range DNA interactions (Fig. 1a; Additional file 1: Figure S2a). By co-expressing in vivo biotinylated dCas9-CBio and sgRNAs targeting specific CREs, the CRE-regulating long-range DNA interactions were cross-linked, followed by restriction enzyme (DpnII) digestion and proximity ligation of interacting DNA fragments. The ligated chimeric DNA were fragmented by sonication, followed by streptavidin-mediated capture of biotinylated dCas9-targeted CREs. The captured CREs and associated long-range DNA interactions were then reverse cross-linked, purified, and analyzed by pair-end sequencing to identify the interacting DNA sequences (Fig. 1a).

With the significantly improved capture efficiency, we explored whether the CAPTURE2.0 system may enable high-resolution and multiplexed analysis of several CREs within the same enhancer cluster in a single experiment. We focused on the locus control region (LCR) of the human β-globin gene cluster consisting of five DNase I hypersensitive sites (DHS) (HS1 to HS5; Fig. 1b). We designed pooled sgRNAs containing two sgRNAs for each DHS (total 10 sgRNAs) and co-expressed with dCas9-CBio in K562 cells (Fig. 1b; Additional file 2: Table S1). The captured chromatin was analyzed by multiplexed CAPTURE-3C-seq to determine LCR-mediated long-range DNA interactions (Fig. 1a; see the “Methods” section). As important quality controls, we analyze genome-scale dCas9 binding by CAPTURE-ChIP-seq in cells expressing target-specific (sgLCR containing pooled 10 sgRNAs) or non-targeting (sgGal4) control sgRNAs. We observed that the sgRNA-targeted DHS regions (HS1 to HS5) were the top enriched peaks in sgLCR-expressing samples, whereas no or minimal enrichment of the predicted sgRNA off-targets was detected (Fig. 1c; Additional file 3: Table S2). Analysis of gene expression using RNA-seq in cells expressing sgLCR or no sgRNA (WT) also revealed minimal changes in transcriptomics (Fig. 1d). Further, the expression of β-globin mRNAs remained unchanged in cells expressing no sgRNA, non-targeting sgGal4, or target-specific sgLCR (Fig. 1d; Additional file 1: Figure S1c), suggesting that the multiplexed capture of LCR enhancers by biotinylated dCas9 did not interfere with endogenous gene expression.

By multiplexed CAPTURE-3C-seq, we identified a total of 2564 LCR-associated long-range interactions, including 1829 (71.8%) interactions within 1 Mb from LCR and 1557 (61.2%) within the β-globin cluster (Fig. 1e; Additional file 4: Table S3). We quantitatively analyze long-range DNA interactions by the FDR-controlled Bayes factor (BF) and identified “high-confidence interactions” with BF scores ≥20 (see the “Methods” section; Additional file 1: Figure S2a). Notably, the interaction frequencies were significantly higher between LCR enhancers and the active genes (*HBE1*, *HBG1*, and *HBG2*) than the repressed gene (*HBD* and *HBB*), consistent with enhancer-promoter loop formation in transcriptional activation [28, 29]. Comparing with CTCF and RNAPII ChIA-PET data [30, 31], we identified known CTCF- or RNAPII-mediated chromatin interactions including the interactions between the flanking HS5 and 3′HS1 insulators (Fig. 1e). We next resolved the captured LCR-mediated interactions (sgLCR) to individual enhancers by retaining the identified pair-end tags (PETs) at each HS enhancer (HS1 to HS5; Fig. 1e). We observed striking similarities in the interaction profiles at each HS enhancer by multiplexed capture compared to our previous results using individual enhancer-specific sgRNAs in independent capture experiments [18] (Additional file 1: Figure S3). These results suggest that the multiplexed capture retains the native chromatin interactions and is capable of capturing high-resolution interactions at multiple CREs in a single experiment. Moreover, by multiplexed capture of β-globin LCR enhancers, we also confirmed our previous findings that the HS3 enhancer contained significantly more long-range interactions than the nearby HS2 enhancer [18] (Fig. 1e; Additional file 1: Figure S3), although HS2 had stronger enhancer functions in reporter assays or transgenic mouse models [32, 33]. Given that all sgRNAs were co-expressed with dCas9 in the same experiment, these results illustrate that the multiplexed capture of multiple constituent enhancers within the same enhancer cluster helps identify the underlying organizational structures controlling enhancer function. Finally, by comparing the normalized number and frequency of long-range DNA interactions identified by CAPTURE, ChIA-PET [30, 31], 4C [34], H3K27ac HiChIP [35], and Hi-C [13, 36], we observed that the multiplexed CAPTURE2.0 system displayed comparable or slightly higher percentage of unique PETs and on-target enrichment than CAPTURE1.0 (Additional file 1: Figure S4a). Both CAPTURE systems outperformed ChIA-PET or Hi-C by increasing the percentage of unique PETs and on-target enrichment (Additional file 1: Figure S4a,b).

Together, the proof-of-principle analyses of β-globin LCR by dCas9 capture not only validated previously identified CRE-mediated long-range DNA interactions, but also established the redesigned CAPTURE2.0 system for high-resolution and multiplexed analysis of locus-specific chromatin interactions in situ.

### Multiplexed CAPTURE of erythroid super-enhancers

**Fig. 2:**
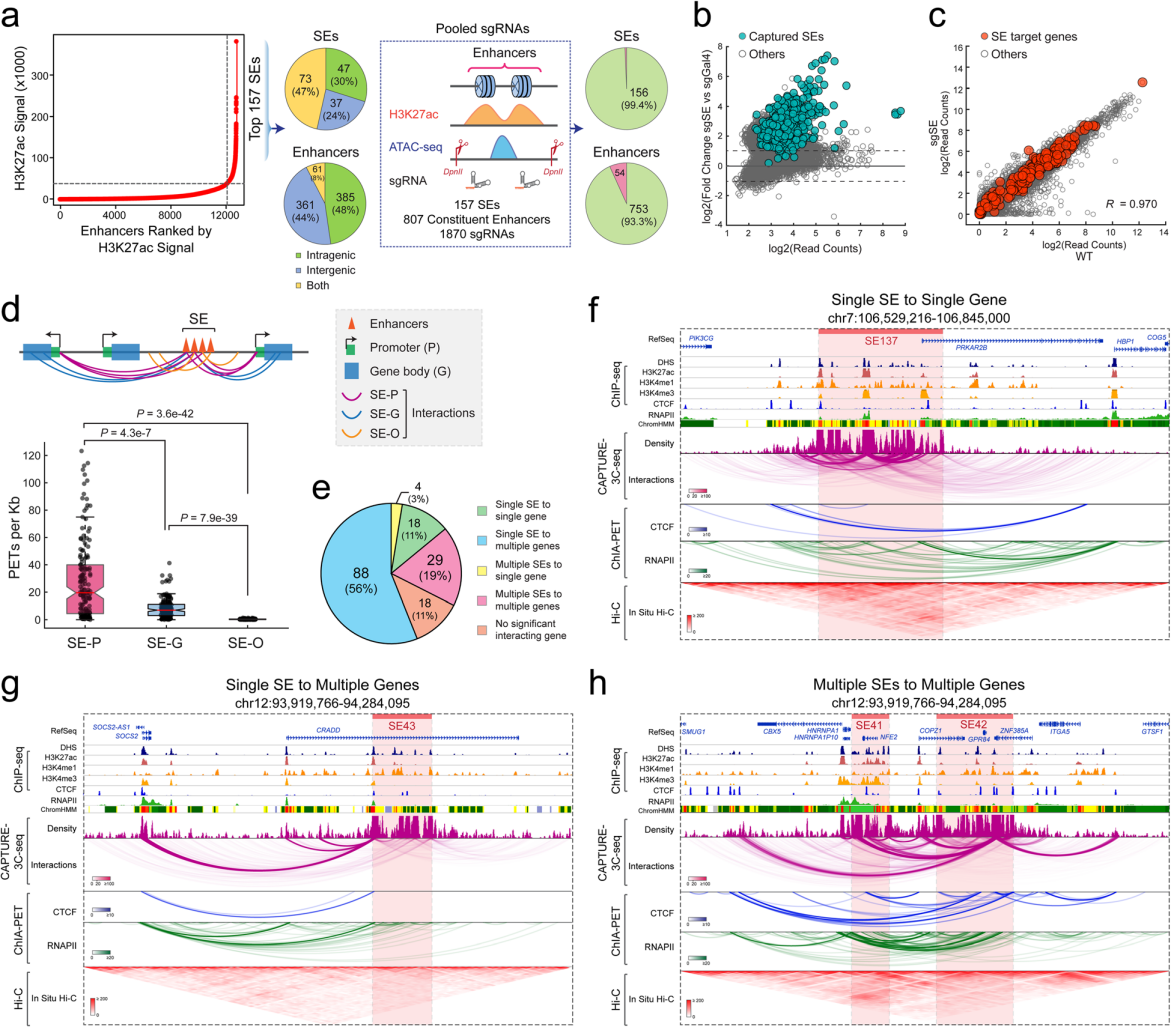
Multiplexed CAPTURE of erythroid super-enhancers. **a** Schematic of the multiplexed analysis of erythroid SEs. SEs were identified by ROSE [37] using H3K27ac ChIP-seq signal in K562 cells. Schematic of sgRNA design is shown. Pie charts on the left show the distribution of the captured SEs and constituent enhancers at intragenic, intergenic, or both regions. Pie charts on the right show the numbers and percentages of captured SEs and constituent enhancers (green color). **b** Genome-wide analysis of dCas9 binding in cells expressing SE-targeting sgRNAs (sgSE) or non-targeting sgGal4. Data points for the sgRNA captured SEs are shown as green. The *x*- and *y*-axes denote the log2 mean read counts and the log2 ratio of read counts in sgSE and sgGal4 samples from *N* = 2 and 4 CAPTURE-ChIP-seq experiments, respectively. **c** Genome-wide differential gene expression analysis was performed using RNA-seq in K562 cells expressing dCas9-CBio with sgSE or WT K562 cells. Data points for SE target genes and other genes are shown as red and gray, respectively. Pearson correlation coefficient (*R*) value is shown (*N* = 2 RNA-seq experiments). **d** Analysis of SE-mediated long-range interactions by categorizing all interactions into SEs to gene promoters (SE-P), SEs to gene bodies (SE-G), and SEs to other genomic regions (SE-O). Schematic of SE-mediated interactions is shown on the top. The interaction frequency was calculated by the normalized PETs per kilobase of captured DNA sequences. Boxes show the median of the data and quartiles, and whiskers extend to 1.5× of the interquartile range. *P* values were calculated by a two-sided Kolmogorov-Smirnov (K-S) test. **e** Pie chart shows the fractions of the captured SE-mediated interactions between SEs and gene targets. **f** A representative locus is shown for the single SE to single gene interactions (SE137). Contact profiles including the density map and interactions for the dCas9-captured SE region (red bar) are shown. The statistical significance of interactions was determined by the Bayes factor (BF) and indicated by the color scale bars. DHS, ChIP-seq, ChromHMM, ChIA-PET (CTCF and RNAPII), and in situ Hi-C data are shown for comparison. **g** A representative locus is shown for the single SE to multiple genes (SE43). **h** A representative locus is shown for the multiple SEs to multiple genes (SE41 and SE42)

Super-enhancers (SEs) are putative enhancer clusters associated with high levels of enhancer activities and enhancer-regulating chromatin factors [37], although the organizational principles of SEs remain largely unknown. A major challenge of analyzing SE structure-function is the lack of sufficient resolution by Hi-C or ChIA-PET-based analysis of SE-mediated chromatin interactions. We reasoned that multiplexed analysis of SEs and their constituent enhancers by high-resolution dCas9 capture in their native chromatin may allow the dissection of spatial organization of SE structure-function in gene regulation. To this end, we designed pooled sgRNAs consisting of 2 or 3 sgRNAs at each constituent enhancer within the top 157 SEs identified by H3K27ac ChIP-seq in K562 cells (total 1870 sgRNAs for 807 constituent enhancers; Fig. 2a; Additional file 2: Table S1) [37]. To avoid interference with endogenous enhancer activity or gene transcription, we designed sgRNAs in close proximity to but not overlapping with the enhancer-associated accessible chromatin by ATAC-seq (Fig. 2a). We next performed multiplexed CAPTURE-3C-seq of dCas9-captured SEs in a single experiment (two biological replicates; Additional file 3: Table S2) and identified high-confidence long-range DNA interactions at 156 of 157 (99.4%) SEs and 753 of 807 (93.3%) individual enhancers (Fig. 2a), indicating a high capture efficiency by multiplexed analysis of hundreds of independent CREs. We also observed significant enrichment of the vast majority of sgRNA-targeted enhancers by CAPTURE-ChIP-seq in cells expressing target-specific (sgSE) related to non-targeting (sgGal4) sgRNAs (Fig. 2b). No significant change in the expression of SE-associated genes, defined as the nearest neighbor genes within a 50-kb genomic region, was detected by RNA-seq (Fig. 2c), suggesting that the multiplexed capture of erythroid SEs by dCas9 did not interfere with endogenous gene transcription.

Enhancers often regulate gene transcription over long distances; thus, it remains difficult to identify the target gene(s) of a given enhancer. By multiplexed capture of chromatin interactions associated with SEs and their constituent enhancers, we sought to determine SE-regulating target genes that physically interact with the capture enhancers. To this end, we categorized the captured SE-mediated long-range DNA interactions into three groups including SEs to gene promoters (SE-P), SEs to gene bodies (SE-G), and SEs to other genomic regions (SE-O) (Fig. 2d). We observed that the frequencies of SE-P interactions are significantly higher than SE-G or SE-O interactions (Fig. 2d). We then designated SE target genes as genes that significantly interact with the capture SEs (see the “Methods” section). By this analysis, we identified 5 distinct patterns of SE-gene interactions, including single SE to single gene, single SE to multiple genes, multiple SEs to single gene, multiple SEs to multiple genes, and SEs containing no significant interacting gene (Fig. 2e). Of note, the most predominant interacting patterns are the single SE to multiple genes (88 SEs or 56%) and multiple SEs to multiple genes (29 or 19%), suggesting that the majority of SEs form long-range interactions with multiple target genes. Compared with CTCF- or RNAPII-mediated interactions by ChIA-PET [30, 31] or in situ Hi-C [13], the multiplexed capture displayed improved resolution at the capture loci (Fig. 2f–h; Additional file 1: S5a-d). These results demonstrate that multiplexed CAPTURE-3C-seq enables the high-resolution analysis of enhancer-mediated chromatin looping and the identification of SE-associated gene targets at the single enhancer resolution.

### Spatial and hierarchical organization of super-enhancers

**Fig. 3:**
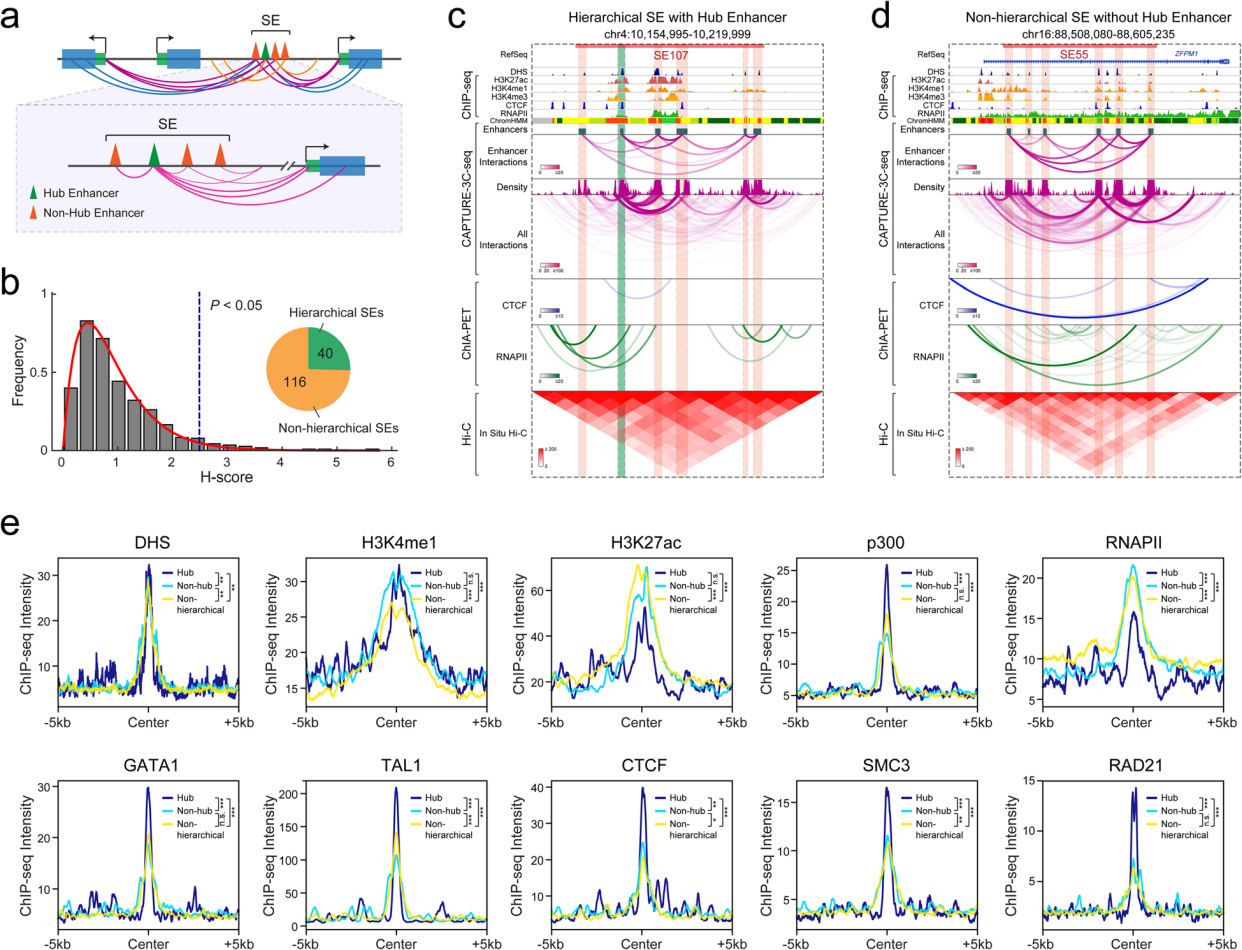
Hierarchical organization of super-enhancers identified by multiplexed CAPTURE. **a** Schematic of the hierarchical structure of SEs based on constituent enhancer-mediated long-range chromatin interactions. **b** Identification of hierarchical SEs by the H-score computational metric. **c** A representative locus is shown for a hierarchical SE containing the hub enhancer. Contact profiles including the density map, interactions between enhancers, and all interactions for the dCas9-captured SE region (red bar) are shown. The identified hub and non-hub enhancers are depicted by green (hub) and red (non-hub) lines, respectively. **d** A representative locus is shown for a non-hierarchical SE without hub enhancer. **e** Chromatin landscapes at hub, non-hub enhancers, and non-hierarchical SEs in K562 cells. Spatial distribution of DHS, histone marks (H3K4me1 and H3K27ac), TFs (p300, GATA1, and TAL1), RNAPII, and chromatin structure factors (CTCF, SMC3, and RAD21) is shown in hub (*N* = 42) and non-hub enhancers (*N* = 260) and non-hierarchical SEs (*N* = 117). *P* values were calculated using the two-sample Kolmogorov-Smirnov (K-S) test. ***P < 0.001, **P < 0.01, *P < 0.05, n.s. not significant

With the significantly improved resolution in identifying locus-specific chromatin interactions by multiplexed CAPTURE-3C-seq, we next determined the spatial organization of SEs by analyzing long-range interactions associated with individual constituent enhancers (Fig. 3a). We previously analyzed SE hierarchy based on Hi-C interactions and identified a subset of SEs containing “hub enhancers” in several human cell lines [38]. We defined hub enhancers using a computational metric called hierarchical score (H-score), in which the frequency of chromatin interactions identified by Hi-C was standardized and compared across the binned human genome. However, due to the limited sequencing depths of the available Hi-C experiments, our analysis of SE hierarchy was limited by a 5-kb resolution [38]. With the high-resolution capture of SE-mediated interactions by CAPTURE-3C-seq (Fig. 2a), we adapted the H-score computational metric by the following modifications (Additional file 1: Figure S2b). First, we counted PETs identified at each enhancer and normalized by enhancer peak size and sequencing depth. The normalized PETs reflect the standardized chromatin interactions at captured enhancers that are independent of capture efficiency by different sgRNAs (Fig. 2b). Second, we calculate the H-score by the mean of PETs of all enhancers within a SE. By this analysis, a higher H-score indicates that the chromatin interactions at the associated enhancer are more enriched than other enhancers within a SE. Third, the H-scores of all enhancers are fitted as gamma distribution, and the enhancers associated with significantly higher H-scores (*P* < 0.05) are referred to as hub enhancers, whereas other enhancers are termed non-hub enhancers (Fig. 3b; Additional file 1: Figure S2b). Lastly, a SE is categorized as a hierarchical SE if it contains at least one hub enhancer, otherwise as a non-hierarchical SE (Fig. 3b).

We applied this pipeline to dissect SE hierarchy using chromatin interactions from multiplexed CAPTURE-3C-seq in K562 cells, and identified 40 of 156 (25.6%) captured SEs that displayed hierarchical structures containing at least one hub enhancer (Fig. 3b; Additional file 5: Table S4; Additional file 6: Table S5). Importantly, some hierarchical SEs were not identified in previous Hi-C-based analysis likely due to the limited resolution [38]. By comparing chromatin interactions identified by ChIA-PET [30, 31], Hi-C [13], and multiplexed CAPTURE at multiple individual hierarchical and non-hierarchical SEs, we observed higher resolution interaction profiles at the captured enhancers by CAPTURE (Fig. 3c, d; Additional file 1: Figure S6a, b), illustrating that the multiplexed dCas9 capture enables quantitative analysis of the spatial organization of individual enhancers within the SE clusters.

To determine the distinguishing features associated with hub and non-hub enhancers within hierarchical SEs, we analyzed the spatial patterns of DHS, enhancer-associated histone marks (H3K4me1 and H3K27ac), and chromatin occupancy of various transcription factors (TFs) (p300, RNAPII, GATA1, and TAL1) and chromatin regulators (CTCF, SMC3, and RAD21). Active enhancers are operationally defined by the presence of H3K4me1 and H3K27ac [39–41]; however, no significant difference in H3K4me1 and H3K27ac ChIP-seq signals was observed at hub and non-hub enhancers, suggesting that hub enhancers cannot be reliably identified by enhancer-associated histone marks. Instead, we observed significantly higher signals for p300 and erythroid master regulators GATA1 and TAL1 and a modest increase in DHS signals at hub enhancers (Fig. 3e). Most importantly, we observed significantly increased binding of CTCF and cohesin subunits (SMC3 and RAD21), two factors essential for mediating long-range chromatin looping and enhancer-promoter interactions [42, 43], at hub enhancers (Fig. 3e). Although non-hub enhancers and non-hierarchical SEs were also enriched with CTCF/cohesin relative to the flanking genomic regions, no or modest differences in CTCF/cohesin binding were observed between non-hub enhancers and non-hierarchical SEs (Fig. 3e). These results are consistent with the role of CTCF and cohesin in mediating chromatin interactions at hub enhancers [38], illustrating that the analysis of high-resolution chromatin interactions by dCas9 capture provides opportunities to examine the underlying organizational principles of SEs in gene regulation. Furthermore, while similar TF motifs were found to be enriched at hierarchical and non-hierarchical SEs (Additional file 1: Figure S6c), we noted that the distances between hierarchical SEs and their associated gene targets were significantly greater than the distances between non-hierarchical SEs and gene targets (*P* = 1.95E−5 by Student’s *t* test; Additional file 1: Figure S6d), suggesting that the hub enhancer-containing hierarchical SEs tend to locate more distal to their gene targets.

### Multiplexed CAPTURE of promoter-centric chromatin interactions

**Fig. 4:**
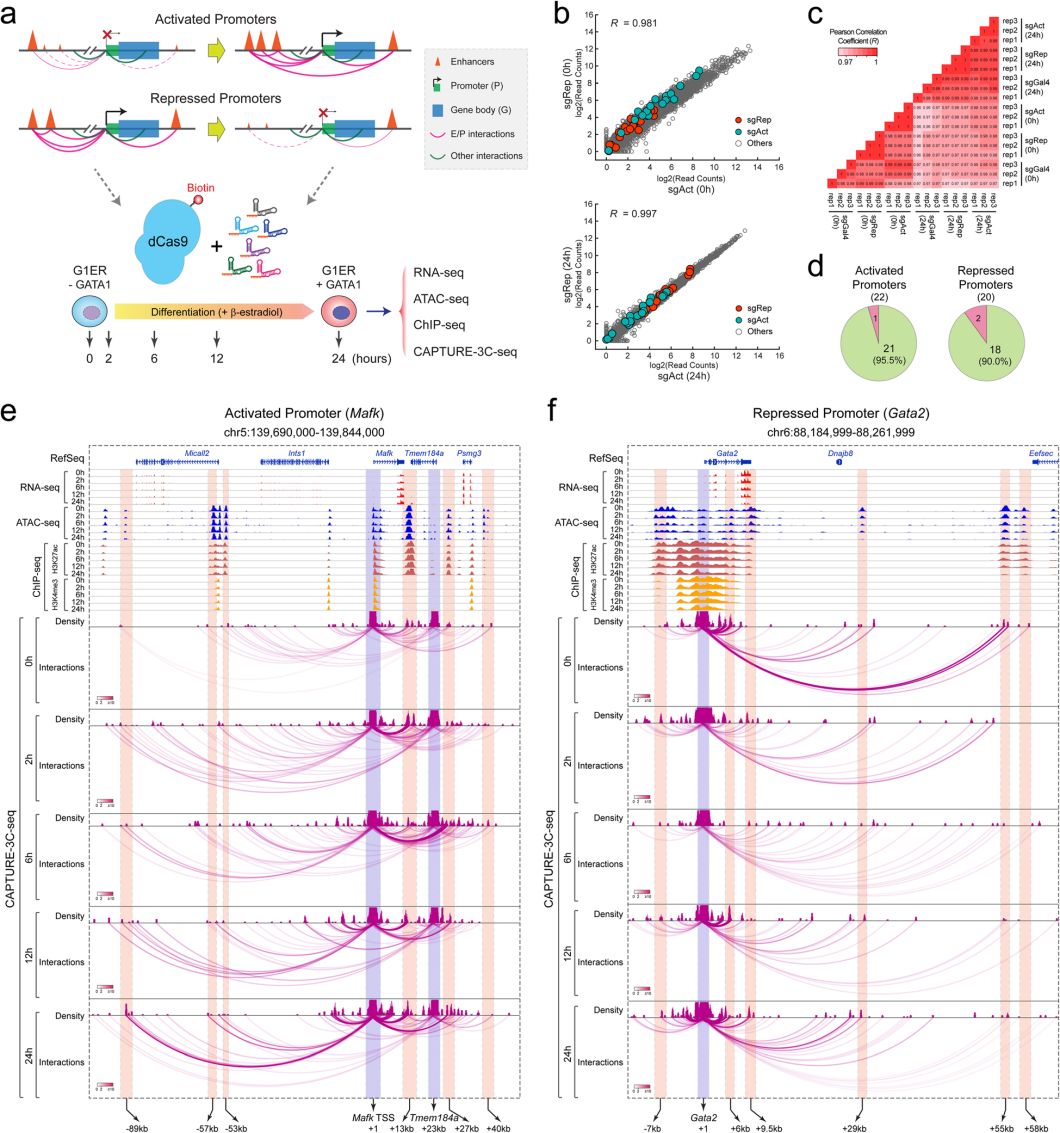
Analysis of promoter-centric chromatin interactions by multiplexed CAPTURE. **a** Schematic of multiplexed CAPTURE of promoter-centric interactions during erythroid differentiation of G1ER cells using sgRNAs targeting 22 activated (sgAct) or 20 repressed (sgRep) promoters. RNA-seq, ATAC-seq, ChIP-seq (H3K27ac and H3K4me3), and CAPTURE-3C-seq of promoter-centric interactions were performed in undifferentiated (0 h) and differentiated (2, 6, 12, and 24 h) G1ER cells. **b** Genome-wide gene expression analysis of cells expressing target-specific sgRNAs (sgAct or sgRep) in undifferentiated (0 h) and differentiated (24 h) G1ER cells. Data points for the sgRNA-targeted activated or repressed promoters are shown as green and red, respectively. The *x*- and *y*-axes denote the log2 read counts in sgAct and sgRep samples from *N* = 3 RNA-seq experiments, respectively. **c** Differential gene expression analysis in G1ER cells expressing dCas9-CBio with sgAct, sgRep, or non-targeting sgGal4. Pearson correlation coefficient (*R*) values are shown for pair-wise comparisons (*N* = 3 RNA-seq experiments). **d** Pie charts show the numbers and percentages of captured activated or repressed promoters (green color). **e** A representative locus is shown for the activated promoter (*Mafk*)-mediated chromatin interactions during erythroid differentiation. Contact profiles including the density map and interactions for the dCas9-captured region (blue bar) and interacting CREs (red bar) are shown. The statistical significance of interactions by the Bayes factor (BF) is indicated by the color scale bars. The locations of the annotated *Mafk* TSS, *Tmem184a* TSS, and candidate enhancers are shown on the bottom. Numbers represent the distances to the *Mafk* TSS (+ 1). Two independent experiments were performed for RNA-seq, ATAC-seq, ChIP-seq, and multiplexed CAPTURE-3C-seq in G1ER cells at varying time points of erythroid differentiation, and the replicates were merged for the genome browser view. **f** A representative locus is shown for the repressed promoter (*Gata2*)-mediated chromatin interactions during erythroid differentiation. The locations of the annotated *Gata2* TSS (+ 1) and candidate enhancers are shown on the bottom

Lineage differentiation requires coordinated control of gene-proximal promoters and distal CREs such as enhancers, yet the temporal dynamics of CRE-mediated chromatin interactions and how they relate to gene activity remain largely unknown. To gain insights into the temporal regulation of locus-specific chromatin interactions during lineage differentiation, we mapped promoter-centric chromatin architecture in the well-established G1E-ER4 (hereafter called G1ER) erythroid cell differentiation model [44]. The *Gata1*-null G1ER cells, a G1E subclone that constitutively expresses an estradiol-activated form of GATA1 fused to the estrogen receptor ligand binding domain (GATA1-ER) [44–46], are maintained in an undifferentiated state and express a high level of GATA2. Upon activation of the GATA1-ER transgene by β-estradiol treatment in G1ER cells, GATA1 binds to its chromatin targets to modulate gene transcription whereas GATA2 expression is sharply downregulated resulting in a “GATA switch” and erythroid differentiation [44, 47, 48]. Notably, while β-estradiol treatment had no apparent effect on GATA1-ER transgene expression, GATA1 shifted its chromatin occupancy during erythroid maturation of G1ER cells (Additional file 1: Figure S7a, b). Using this model, we sought to determine how promoter-mediated chromatin loops are established or reconstructed upon gene activation or deactivation, respectively, at varying time points of GATA1-induced erythroid differentiation (0, 2, 6, 12, and 24 h) (Fig. 4a). The differentiation-associated changes in gene expression, chromatin accessibility, epigenetic landscapes, and promoter-centric chromatin interactions were determined by RNA-seq, ATAC-seq, ChIP-seq, and multiplexed CAPTURE-3C-seq, respectively (Fig. 4a; Additional file 2: Table S1).

We designed pooled sgRNAs consisting of 2 or 3 sgRNAs per promoter region for 22 promoters of the most significantly upregulated genes (hereafter called “activated” promoters) during erythroid differentiation of G1ER cells (Fig. 4a). Likewise, we designed pooled sgRNAs consisting of 2 or 3 sgRNAs per promoter region for 20 promoters of the most significantly downregulated genes (hereafter called “repressed” promoters). To avoid interference with endogenous promoter activity or gene transcription, we designed sgRNAs in close proximity to but not overlapping with the promoter-associated accessible chromatin. As important quality controls, we performed RNA-seq in cells expressing sgRNAs targeting activated promoters (sgAct), repressed promoters (sgRep), or non-targeting sgGal4 in undifferentiated (0 h) and differentiated (24 h) G1ER cells. The global transcriptomic profiles were highly similar in G1ER cells expressing target-specific (sgAct or sgRep) or non-targeting sgGal4 before or after differentiation (Fig. 4b, c; Additional file 1: Figure S7c-f). The expression of the captured promoter-associated genes remained unchanged between different groups in undifferentiated and differentiated G1ER cells, respectively, suggesting that the multiplexed capture of promoter-centric interactions by dCas9 did not interfere with endogenous gene transcription.

By multiplexed CAPTURE-3C-seq of dCas9-captured promoters in G1ER cells, we identified significant long-range DNA interactions at 21 of 22 (95.5%) activated promoters and 18 of 20 (90.0%) repressed promoters (Fig. 4d; Additional file 7: Table S6), respectively, illustrating a high capture efficiency comparable to multiplexed enhancer capture (Fig. 2a). We next compared the high-resolution chromatin interaction profiles with gene expression (by RNA-seq), chromatin accessibility (by ATAC-seq), and histone modifications (H3K27ac and H3K4me3 by ChIP-seq) at various time points of GATA1-induced erythroid differentiation (0 to 24 h) at multiple independent loci. Importantly, we noted strong correlations between promoter-mediated long-range DNA interactions and gene expression at both activated and repressed promoters (Fig. 4e, f; Additional file 1: Figure S8a-e). For instance, GATA1 activation by β-estradiol treatment led to significant and progressive activation of *Mafk* and *Tmem184a* genes in G1ER cells (Fig. 4e; Additional file 1: Figure S8a, b), consistent with rapid gain of chromatin interactions between the captured *Mafk* and *Tmem184a* promoters and several annotated enhancers (− 89 kb, + 13 kb, + 27 kb, and + 40 kb; Fig. 4e) after 2 h of GATA1 activation. The enhancer-promoter interactions progressively gained strength during subsequent differentiation (6 to 24 h). By contrast, little or no change in chromatin accessibility or epigenetic landscapes was noted at the captured promoters and their interacting enhancers at the same time points (Fig. 4e). At the repressed promoters, the GATA1-induced erythroid differentiation resulted in a significant and progressive downregulation of GATA2 mRNA expression (Additional file 1: Figure S8c). *Gata2* silencing was associated with significant and rapid loss of chromatin interactions between the captured *Gata2* promoter and some but not all enhancers as early as 2 h of GATA1 activation, whereas modest changes in chromatin accessibility and epigenetic landscapes at *Gata2* promoter and enhancers were noted at later time points (12 h and 24 h) (Fig. 4f). Similar patterns were observed at the other representative activated (*Btn1a1*) and repressed (*Ms4a2*) promoters (Additional file 1: Figure S8d,e).

Together, the side-by-side comparisons between chromatin interactions, gene expression, and epigenetic changes at multiple loci demonstrate that changes in gene activation or repression are strongly associated with reconstruction of promoter-mediated chromatin interactions, in particular enhancer-promoter loop formation, during lineage differentiation. Furthermore, the refined kinetic analyses at several independent loci demonstrate that changes in gene expression occur in close cooperation with gained or lost enhancer-promoter interactions and precede significant changes in epigenetic landscapes or chromatin accessibility, supporting the notion that enhancer-promoter loop formation causally underlies gene activation or deactivation [18, 28, 29].

### Temporal dynamics of enhancer-promoter interactions during differentiation

**Fig. 5:**
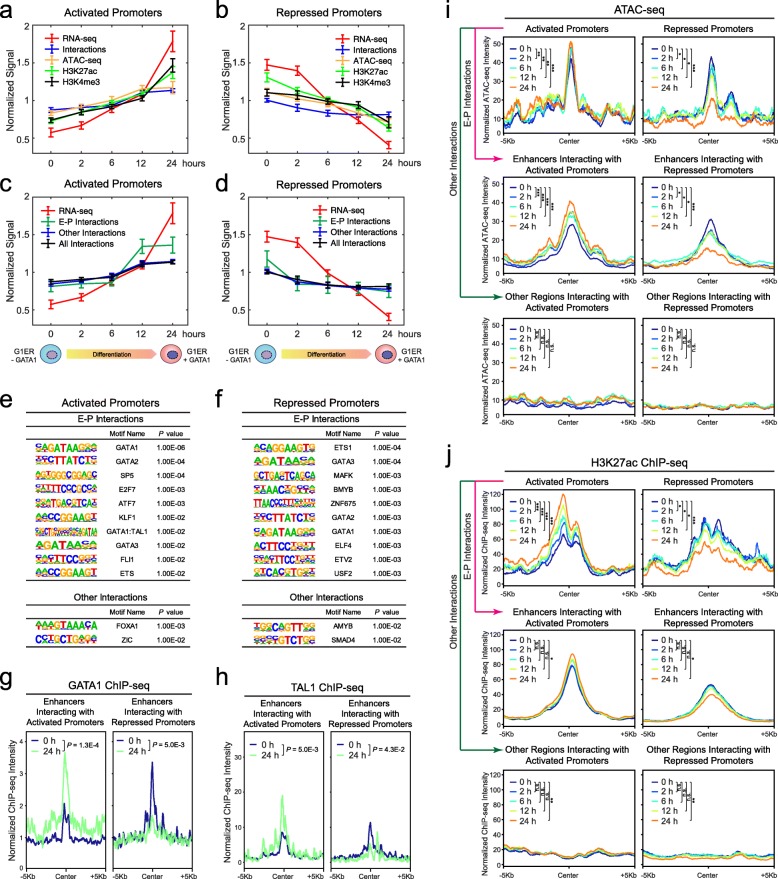
Temporal dynamics of promoter-centric interactions during differentiation. **a** Temporal changes in gene expression (RNA-seq), chromatin interactions (CAPTURE-3C-seq), chromatin accessibility (ATAC-seq), and epigenetic landscapes (H3K27ac and H3K4me3 ChIP-seq) at the captured activated promoters during erythroid differentiation of G1ER cells. The *x*-axis denotes the time points of undifferentiated (0 h) or differentiated (2, 6, 12, and 24 h) G1ER cells. The *y*-axis denotes the normalized signals calculated by the mean normalized gene FPKM (RNA-seq), read counts (ATAC-seq and ChIP-seq), or PETs (CAPTURE-3C-seq) per kilobase of captured genomic region per million mapped reads and shown as mean ± SEM (*N* = 22 activated promoters). **b** Temporal changes in gene expression, chromatin interactions, chromatin accessibility, and epigenetic landscapes at the captured repressed promoters during erythroid differentiation of G1ER cells. The *y*-axis denotes the normalized signals shown as mean ± SEM (*N* = 20 repressed promoters). **c** Temporal changes in gene expression and chromatin interactions (all, E-P, and other interactions) at the captured activated promoters during erythroid differentiation. E-P interactions and gene expression were significantly and positively correlated (Pearson correlation coefficient *R* = 0.890, *P* = 0.022 by Student’s *t* test), whereas the other interactions showed no significant correlation (*R* = 0.698, *P* = 0.095). **d** Temporal changes in gene expression and chromatin interactions (all, E-P, and other interactions) at the captured repressed promoters. E-P interactions and gene expression were significantly and positively correlated (Pearson correlation coefficient *R* = 0.905, *P* = 0.018), and the other interactions also showed a positive correlation with expression (*R* = 0.871, *P* = 0.028). **e** Top enriched TF motifs at genomic regions associated with E-P interactions or other interactions with the captured activated promoters. **f** Top enriched TF motifs at the genomic regions associated with E-P interactions or other interactions with the captured repressed promoters. **g** Chromatin occupancy of GATA1 at enhancers interacting with the captured activated or repressed promoters in undifferentiated (0 h) and differentiated (24 h) G1ER cells. *P* values were calculated using the two-sample Kolmogorov-Smirnov (K-S) test. **h** Chromatin occupancy of TAL1 at enhancers interacting with the captured activated or repressed promoters in undifferentiated (0 h) and differentiated (24 h) G1ER cells. **i** Spatial distribution of chromatin accessibility at the captured activated or repressed promoters, enhancers, and other regions interacting with captured promoters in undifferentiated (0 h) and differentiated (2, 6, 12, and 24 h) G1ER cells. *P* values were calculated using the two-sample Kolmogorov-Smirnov (K-S) test. ***P < 0.0001, **P < 0.01, *P < 0.05, n.s. not significant. **j** Spatial distribution of H3K27ac ChIP-seq signals at the captured activated or repressed promoters, enhancers, and other regions interacting with captured promoters in undifferentiated and differentiated G1ER cells

Given the strong correlations between promoter-centric chromatin interactions and gene expression at individual loci, we next compared the temporal changes in gene expression, chromatin interactions, accessibility, and epigenetic landscapes at the captured activated and repressed promoters during differentiation. At the global level, we observed progressive gain (at activated promoters) or loss (at repressed promoters) of chromatin interactions, ATAC-seq, H3K27ac, and H3K4me3 ChIP-seq signals associated with gene activation or repression, respectively (Fig. 5a, b; Additional file 1: Figure S9a, b). We next categorized the captured promoter-centric interactions into enhancer-promoter (E-P) interactions and other interactions based on the overlap between the captured PETs and annotated enhancers (Additional file 1: Figure S2c; Additional file 8: Table S7; see the “Methods” section). By this analysis, we noted that the E-P interactions are more significantly and positively correlated with gene expression changes at both activated and repressed promoters (Pearson correlation coefficient *R* = 0.890 and 0.905, *P* = 0.022 and 0.018 by Student’s *t* test, respectively; Fig. 5c, d). Consistent with the global analysis, the progressive gain or loss of E-P interactions is the most significantly changed chromatin feature at multiple individual loci upon gene activation or repression during differentiation (Fig. 4e, f; Additional file 1: Figure S8d, e), respectively.

To determine the underlying TFs associated with the dynamically regulated chromatin interactions during erythroid differentiation, we performed motif enrichment analysis of genomic regions associated with E-P or other interactions (Fig. 5e, f). We identified TF motifs for hematopoietic lineage TFs including GATA factors (GATA1 and GATA2), TAL1, and KLF1 as the top enriched motifs associated with E-P interactions at the activated promoters. There are few enriched motifs associated with other interactions (Fig. 5e). Similarly at the repressed promoters, motifs for lineage master TFs such as GATA factors and BMYB are highly associated with E-P interactions but not with other interactions (Fig. 5f). The enrichment of TAL1::GATA1 composite motif in E-P interactions at activated but not repressed promoters is consistent with the role of GATA1/TAL1 complex in transcriptional activation during erythroid differentiation [49, 50]. The enrichment of motifs for GATA factors is consistent with the roles of GATA1 and GATA2 as both transcriptional activators and repressors in hematopoietic cells [44, 48, 51, 52].

We then investigated whether the binding of GATA1 and TAL1 underlies the gain or loss of E-P interactions during erythroid differentiation by ChIP-seq of GATA1 and TAL1 before or after GATA1 activation in G1ER cells (Fig. 5g, h). We noted significantly increased binding of GATA1 at enhancers associated with gained E-P interactions and significantly decreased GATA1 binding at enhancers associated with lost E-P interactions in differentiated G1ER cells (Fig. 5g). Modest increases in GATA1 binding at activated promoters and decreases in GATA1 binding at repressed promoters were also noted in differentiated G1ER cells, respectively (Additional file 1: Figure S10a). Likewise, significantly increased TAL1 binding at enhancers associated with gained E-P interactions and activated promoters were observed (Fig. 5h; Additional file 1: Figure S10b). Furthermore, we noted significant enrichment of CTCF occupancy in the proximity of the captured activated and repressed promoters, as well as the enhancers interacting with the activated promoters (Additional file 1: Figure S10c). These results demonstrate that the chromatin occupancy of CTCF and lineage master TFs strongly associates with the formation or reconfiguration of E-P interactions during lineage differentiation.

Finally, we examined the kinetic changes in epigenetic landscapes at the captured activated or repressed promoters and their interacting genomic regions by ATAC-seq and ChIP-seq during G1ER differentiation (0, 2, 6, 12, and 24 h; Additional file 2: Table S1). We observed strongly coordinated increases in chromatin accessibility and H3K27ac at activated promoters and their interacting enhancers (Fig. 5i, j), indicating cooperated epigenetic changes likely through progressively gained E-P interactions (Fig. 5a, c). Likewise, we observed coordinated decreases in chromatin accessibility and H3K27ac at repressed promoters and their interacting enhancers (Fig. 5i, j), consistent with progressively lost E-P interactions (Fig. 5b, d). Slight or modest changes in H3K4me3 at the capture promoters were also noted (Additional file 1: Figure S10d), consistent with the transcriptional changes associated with altered promoter activities during erythroid differentiation.

Taken together, the refined kinetic analyses of promoter-centric interactions, gene expression, and epigenetic changes at single locus and global levels illustrate that gene activation or repression is strongly associated with the reconfiguration of E-P looping during lineage differentiation. The genomic regions associated with gained or lost E-P interactions are enriched with binding sites for lineage master TFs including GATA1 and TAL1, and changes in GATA1 and TAL1 chromatin occupancy strongly associate with the reorganized E-P interactions. Therefore, our findings support an instructive role of E-P interactions in controlling gene transcription in response to lineage master TF-induced differentiation, and the reconfiguration of E-P loop formation causally underlies gene activation or deactivation during development. Furthermore, our results illustrate the ability of the redesigned dCas9-based CAPTURE for high-throughput analysis of the spatial organization and temporal dynamics of locus-specific chromatin interactions, providing opportunities for interrogating the organizational principles of genome structure and function.

## Discussion

### Multiplexed CAPTURE of enhancer hierarchical structure

It is estimated that the human genome contains more than one million CREs that control tissue and developmental stage-specific gene expression. SEs were described as intensively marked clusters of enhancers that control the expression of cell identity-related genes [37]; however, it remains unclear how SEs are spatially organized through the assembly of their constituent enhancers. Here, we describe the resigned CAPTURE system for high-resolution and multiplexed analysis of a few to hundreds of enhancers in a single high-throughput experiment. Compared to individual capture assays [18, 19], the multiplexed analyses by the redesigned CAPTURE provide several advantages, including (1) the significantly increased capture efficiency; (2) the combination of C-terminal dCas9 biotin-tagging and endogenous biotin ligases enables simplification of cell line engineering, thus broader applications in various cell models including primary cells; and (3) more cost effective for high-resolution and high-throughput mapping of CRE-mediated chromatin interactions in a single experiment. We showcased the applications of the new system by multiplexed capture of the human β-globin LCR and erythroid SEs and revealed distinct patterns of SE-gene interactions and the hierarchical organization of SEs. These results demonstrate that the multiplexed CAPTURE coupled with the 3C approach enables quantitative analysis of enhancer-mediated chromatin looping and identification of SE-associated gene targets at single enhancer resolution. Thus, the CAPTURE approach provides a platform for the systematic dissection of SE constituents and the underlying formative composition controlling enhancer structure-function in a mammalian genome.

### Temporal dynamics of 3D chromatin structure during development

High-order chromatin structures have been analyzed by Hi-C-based approaches in mammalian development [1–3]; however, the quantitative analysis of enhancer-promoter (E-P) interactions remains challenging. This is due in large to insufficient resolution of Hi-C assays and the prohibitive cost for generating Hi-C datasets with basepair resolution [9–16]. By multiplexed capture of promoter-centric interactions in a well-defined model of erythroid differentiation, we revealed the time-resolved, high-resolution E-P interaction profiles during the immediate and late phases of lineage differentiation of G1ER cells. By comparative analyses of changes in gene expression, chromatin accessibility, and epigenetic landscapes, we uncovered that the formation or disruption of E-P interactions is significantly and positively correlated with gene expression changes at both activated and repressed promoters, respectively. The changes in gene expression and chromatin interactions are highly correlated and precede changes in epigenetic landscapes or chromatin accessibility, supporting the causative roles of E-P loop formation in gene transcription [18, 28, 29]. In addition, we found that the developmentally regulated E-P interactions highly associate with chromatin occupancy of lineage-specifying TFs to rewire 3D chromatin interactions during differentiation. These findings support an instructive role of E-P interactions in controlling gene transcription in response to TF-induced differentiation. Furthermore, the unbiased analysis of promoter-centric interactions helps identify the complete set of promoter-interacting constitutive or lineage-specific CREs and will facilitate the identification and follow-up studies of functionally relevant CREs controlling gene expression during lineage differentiation.

### Considerations for multiplexed CAPTURE assays

The successful application of multiplexed capture of locus-specific chromatin interactions requires several important considerations in assay development. First, the sgRNAs should be designed to locate in close proximity to the captured CREs, but not overlapping with known TF binding sites to avoid interference with endogenous protein-DNA interactions. Second, the analysis of CRE-mediated long-range DNA interactions by 3C requires the design of sgRNAs in the same chromatin fragments digested by the restriction enzyme (e.g., DpnII). Multiple sgRNAs may be designed for multiple DpnII-digested fragments containing the captured CREs to maximize the capture efficiency. Third, the genome-wide specificity and on-target enrichment of the pooled sgRNAs should be evaluated to minimize off-target effects, although the PETs associated with non-captured regions may be filtered during data processing to eliminate off-target signals. Fourth, the multiplexed analysis of many CREs in a single experiment requires comparable on-target enrichment for each sgRNA to minimize variations in capture efficiency. Finally, multiplexed capture of CRE-regulating protein factors may be limited due to the difficulty to ascribe the identified *trans*-acting factors to specific CREs; however, it may be used to study larger chromatin domains such as heterochromatin, euchromatin, and lamina-associated domains. The multiplexed capture of interacting proteins at many loci in the same chromatin domains may help identify chromatin regulators that are shared or unique to specific chromatin architectures in controlling genome structure and function.

## Methods

### Cells and cell culture

Human K562 cells were obtained from ATCC and cultured in an IMDM medium containing 10% fetal bovine serum (FBS) and 1% penicillin/streptomycin (P/S). Human HEK293T cells were obtained from ATCC and cultured in a DMEM medium containing 10% FBS and 1% P/S. G1E and G1ER cells were cultured as described [44]. All cultures were incubated at 37 °C in 5% CO_2_. All cell lines were tested negative for mycoplasma contamination. No cell line used in this study was found in the database of commonly misidentified cell lines maintained by ICLAC and NCBI BioSample.

### sgRNA design and cloning

sgRNAs were designed to target the proximity of *cis*-regulatory elements using the public tool (http://crispr.mit.edu/) as previously described [18, 19]. sgRNAs targeting promoters or LCR enhancers were cloned into the U6 promoter-driven lentiviral vector pSLQ1651-sgRNA(F+E)-sgGal4 (Addgene, #100549) by PCR amplification using a common reverse primer and unique forward primers containing the protospacer sequence [53]. The PCR amplicon and the sgRNA vector containing a mCherry reporter were digested by BstXI and XhoI. The digested DNA fragments were then purified, ligated to the digested sgRNA vector, and validated by Sanger sequencing. For multiplexed SE capture, the top 157 SEs were identified by ROSE [37] based on H3K27ac ChIP-seq signals. All regions overlapping with annotated promoters within SEs were removed. For each constituent enhancer, 2–3 sgRNAs were designed targeting the proximity but not overlapping with enhancer-associated accessible chromatin based on ATAC-seq. DNA oligonucleotides containing sgRNAs were synthesized on a programmable microarray using a B3 Synthesizer (CustomArray). Full-length oligonucleotides (96 nt) were amplified for 15 cycles by PCR using Phusion® High-Fidelity DNA Polymerase (referred to as PCR1). PCR2 was then performed using amplicon from PCR1 as the template to remove the barcodes and replace with homology arms of pSLQ1651-sgRNA(F+E)-sgGal4 vector for Gibson assembly reaction. The PCR amplicon was gel purified. The pSLQ1651-sgRNA(F+E)-sgGal4 vector was digested by BstXI and XhoI and gel purified. Gibson assembly was performed to generate the sgRNA vector pool following the manufacturer’s protocol. Briefly, 10 μl of the Gibson assembly reaction was added to 100 μl *E. cloni* 10G ELITE electrocompetent cells (Lucigen, #60052-4) and electroporated using Bio-Rad MicroPulser. The transformed bacteria were plated onto pre-warmed 24.5-cm^2^ bioassay plates with ampicillin and incubated overnight at 37 °C. The colonies were counted to calculate the library coverage (> 200×). All colonies were collected, and maxiprep was performed to isolate the sgRNA vectors. A pool of lentiviruses containing all sgRNAs was produced as previously described [18, 19]. The sequences of all sgRNAs used in this study are listed in Additional file 2: Table S1.

### Cloning of CAPTURE vectors

To generate the pLVX-EF1a-BirA-P2A-FB-dCas9-IRES-zsGreen1 (CAPTURE1.1) vector, the BirA-V5-6xHis and FB-dCas9 were amplified from the pEF1a-BirA-V5-neo vector (Addgene, #61357) and pEF1a-FB-dCas9-puro (Addgene, #100547) as templates, respectively, and cloned into XbaI-digested pLVX-EF1a-IRES-zsGreen1 by In-Fusion® HD Cloning Kit (Clontech). To construct the pLVX-EF1a-NBio-dCas9-IRES-zsGreen1 (CAPTURE2.0-NBio) and pLVX-EF1a-dCas9-CBio-IRES-zsGreen1 (CAPTURE2.0-CBio) vectors, the dCas9 and BioTAP sequences were amplified from the pEF1a-FB-dCas9-puro (Addgene, #100547) and pEF1a-FB-dCas9-bioTAP-puro, respectively. Then the PCR amplicons were subcloned into XbaI-digested pLVX-EF1a-IRES-zsGreen1 by In-Fusion® HD Cloning Kit (Clontech). The pLVX-EF1a-BirA-P2A-FB-dCas9-IRES-zsGreen1, pLVX-EF1a-NBio-dCas9-IRES-zsGreen1, and pLVX-EF1a-dCas9-CBio-IRES-zsGreen1 vectors were deposited to Addgene under #138417, #138418, and #138419, respectively.

### Lentivirus production and transduction

Lentiviruses containing pLVX-EF1a-BirA-P2A-FB-dCas9-IRES-zsGreen1, pLVX-EF1a-NBio-dCas9-IRES-zsGreen1, pLVX-EF1a-dCas9-CBio-IRES-zsGreen1, or sgRNAs were packaged in HEK293T cells as previously described [18, 19] with modifications. Briefly, 4 μg of pΔ8.9, 2 μg of VSV-G, and 6 μg lentiviral vectors were co-transfected into HEK293T cells in 10-cm dishes. Lentiviruses were collected by harvesting the supernatant 48–72 h after transfection. For CAPTURE1.0, FB-dCas9 and BirA-expressing K562 stable cells [18, 19] were transduced with sgRNA-expressing lentiviruses in 6-well plates. For CAPTURE1.1 and CAPTURE2.0, K562 cells were co-transduced with BirA-P2A-FB-dCas9, NBio-dCas9, or dCas9-CBio and sgRNA-expressing lentiviruses. To maximize sgRNA expression, the top 5% of mCherry-positive cells were sorted 48 h post-transfection.

### RNA-seq and qRT-PCR analysis

RNA was isolated using RNeasy Plus Mini Kit following the manufacturer’s protocol (Qiagen). RNA-seq libraries were prepared using NEBNext Ultra II Directional RNA Library Prep Kit (NEB). Sequencing reads were aligned to human (hg19) or mouse (mm10) reference genome by TopHat v2.0.13 [54] with the parameters: --solexaquals --no-novel-juncs. Uniquely mapped reads were then process by Cufflinks [55] for assembly of gene expression FPKM (Fragments Per Kilobase of transcript per Million mapped reads). Quantitative RT-PCR (qRT-PCR) was performed using the iQ SYBR Green Supermix (Bio-Rad) as previously described [56]. Primer sequences are listed in Additional file 2: Table S1. RNA-seq data in G1E and undifferentiated (0 h) and differentiated (24 h) G1E-ER4 cells were obtained from GEO (GSM2400140, GSM2400141, GSM240018, GSM240019, GSM2400132, and GSM2400133) and aligned to the mm10 (GENCODE Version M4) reference genome. Sequencing reads were processed using the “Long RNA-Seq Processing Pipeline” from ENCODE [30]. Briefly, sequencing reads were aligned using STAR v2.4.0 [57] with ENCODE standard options and expression was quantified using RSEM v1.2.15 [58] using default parameters. Gene expression values as transcripts per million (TPM) were used to compare *Gata1* expression in G1E and G1E-ER4 cells.

### ChIP-seq and data analysis

ChIP was performed as described [18] using antibodies for H3K4me3 (Millipore, #04-745) or H3K27ac (Abcam, #ab4729) in G1ER cells at various time points of differentiation. ChIP-seq libraries were generated using NEBNext Ultra II DNA Library Prep Kit following the manufacturer’s protocol (NEB) and sequenced on an Illumina NextSeq500 system using the 75-bp high output kit. ChIP-seq raw reads were aligned to the mouse genome assembly (mm10) using Bowtie2 [59] with the default parameters. Unique mapped reads were used for peak calling by MACS with the “--nomodel” parameter [60]*.* The read depths and signal density of chromosomal regions were performed and visualized by deepTools2 [61]. To compare ChIP-seq signals at promoters, enhancers, and other genomic regions between samples, we also calculated FPKM (Fragments Per Kilobase of transcript per Million mapped reads) for each region. GATA1 ChIP-seq data in G1E and undifferentiated (0 h) and differentiated (24 h) G1E-ER4 cells were obtained from GEO (GSM946538, GSM923581, GSM995441, GSM995445, GSM995439, and GSM923572). Sequencing reads were aligned to mm10 mouse reference genome using Bowtie2 with default parameters [57]. Uniquely mapped reads were used for peak calling by MACS with the “--nomodel” parameter and with *P* value 10^−8^ [58]. Shared and sample-specific GATA1 peaks were derived using the “intersect” command from BEDTools suite [62].

### ATAC-seq and data analysis

ATAC-seq was performed as previously described with modifications [63]. Briefly, 5 × 10^4^ G1ER cells were washed twice in PBS and resuspended in 500 μl lysis buffer (10 mM Tris-HCl, 10 mM NaCl, 3 mM MgCl_2_, 0.1% NP-40, pH 7.4). Nuclei were harvested by centrifugation at 500×*g* for 10 min at 4 °C. Nuclei were suspended in 50 μl of tagmentation mix (10 mM TAPS, 5 mM MgCl_2_, pH 8.0, and 2.5 μl Τn5) and incubated at 37 °C for 30 min. Tagmentation reaction was terminated by incubating nuclei at room temperature for 2 min followed by incubation at 55 °C for 7 min after adding 10 μl of 0.2% SDS. Tn5 transposase-tagged DNA was purified using QIAquick MinElute PCR Purification kit (Qiagen) and amplified using KAPA HiFi Hotstart PCR Kit (KAPA). ATAC-seq libraries were sequenced on an Illumina NextSeq500 system using the 75-bp high output kit. Raw reads were trimmed to remove adaptor sequence and aligned to mouse genome assembly (mm10) using Bowtie2 [59] with default parameters. Only tags that uniquely mapped to the genome were used for analysis. ATAC-seq peaks were identified using MACS with the “--nomodel” parameter [60]. To compare signals at promoters, enhancers, and other genomic regions between samples, we calculated FPKM (Fragments Per Kilobase of transcript per Million mapped reads) for each region.

### CAPTURE assays

The dCas9-based CAPTURE assays consist of CAPTURE-ChIP-seq, CAPTURE-ChIP-qPCR, CAPTURE-3C-seq experiments, and associated data analysis methods.

#### CAPTURE-ChIP-seq

K562 stable cells co-expressing FB-dCas9 and BirA (CAPTURE1.0 and CAPTURE1.1) or BioTAP-tagged dCas9 (CAPTURE2.0 NBio or CBio) and sequence-specific or non-targeting sgRNAs were used for CAPTURE-ChIP experiments. Cells were cross-linked with 1% formaldehyde for 10 min and quenched with 0.125 M of glycine for 5 min. After washing with PBS, cells were lysed in 1 ml RIPA buffer (10 mM Tris-HCl, 1 mM EDTA, 0.1% sodium deoxycholate, 0.1% SDS, 1% Triton X-100, pH 8.0) and rotated for 15 min at 4 °C. Cell nuclei were collected by centrifugation at 2300×*g* for 5 min at 4 °C. Nuclei were resuspended in 500 μl of 0.5% SDS lysis buffer (0.5% SDS, 10 mM EDTA, 50 mM Tris-HCl, pH 8.0), and chromatin was sonicated to an average size 200 to 500 bp on the Branson Sonifier 450 ultrasonic processor (20% amplitude, 0.5 s on 1 s off for 30 s). Fragmented chromatin was centrifuged at 16,100×*g* for 10 min at 4 °C. Supernatant containing soluble chromatin was transferred to a new tube. Final concentration 300 mM NaCl was added to 450 μl of supernatant, followed by incubation with 10 μl of MyOne Streptavidin T1 Dynabeads (Thermo Fisher Scientific) at 4 °C. After overnight incubation, Dynabeads were washed twice with 1 ml of 2% SDS, twice with 1 ml of RIPA buffer with 0.5 M NaCl, twice with 1 ml of LiCl buffer (250 mM LiCl, 0.5% NP-40, 0.5% sodium deoxycholate, 1 mM EDTA and 10 mM Tris-HCl, pH 8.0), and twice with 1 ml of TE buffer (10 mM Tris-HCl, 1 mM EDTA, pH 8.0). Chromatin was eluted in SDS elution buffer (1% SDS, 10 mM EDTA, 50 mM Tris-HCl, pH 8.0) and reverse cross-linked at 65 °C overnight. ChIP DNA was incubated with RNase A (5 μg/ml) and protease K (0.2 mg/ml) at 37 °C for 30 min and purified using QIAquick Spin columns (Qiagen). 1~10 ng of ChIP DNA was processed for library generation using the NEBNext Ultra II DNA Library Prep Kit (NEB), and sequenced on an Illumina NextSeq500 system using the 75-bp high output kit.

#### CAPTURE-ChIP-qPCR

For CAPTURE-ChIP-qPCR experiments, 1 to 5 × 10^6^ K562 stable cells transduced with target-specific sgRNAs or non-targeting sgGal4 were used. The captured DNA was isolated using the protocol described for CAPTURE-ChIP experiment and analyzed by quantitative PCR (qPCR). For input control samples, 80 μl of SDS elution buffer was added into 20 μl of sonicated soluble chromatin. The samples were reverse cross-linked at 65 °C overnight. DNA fragments were purified with the QIAquick PCR Purification Kit and eluted with 100 μl of EB buffer (Qiagen). Primer sequences are listed in Additional file 2: Table S1.

#### CAPTURE-ChIP-seq data analysis

Raw reads were aligned to human (hg19) or mouse (mm10) genome assembly using Bowtie2 [59] with default parameters. Peak calling was performed by MACS using the “--nomodel” parameter [60]*.* Peaks that overlap with the blacklist regions annotated by the ENCODE project [30], the repeat masked region (chr2:33,141,250-33,142,690; hg19), or the validated non-targeting control sgRNA (sgGal4)-enriched regions (chr6:119,558,373-119,558,873, chr21:15,457,141-15,457,641, chr20:26,188,800-26,190,400, and chr11:192,110-192,410; hg19) were removed. MAnorm [64] was applied to compare ChIP-seq signal intensities in samples prepared from cells expressing the target-specific sgRNAs or non-targeting sgGal4. The window size was 1 kb which matched the average width of the identified ChIP-seq peaks.

#### CAPTURE-3C-seq

CAPTURE-3C-seq assays were performed as previously described [18, 19] with modifications. Specifically, 5 × 10^6^ cells were cross-linked with 2 mM EGS (ethylene glycol bis(succinimidyl succinate)) (Thermo Fisher Scientific) for 45 min and 1% formaldehyde for 10 min and quenched with 0.25 mM of glycine for 5 min at room temperature with rotation. After two washes with PBS, cells were resuspended in 1 ml of ice-cold cell lysis buffer (25 mM Tris-HCl pH 7.4, 85 mM KCl, 0.1% Triton X-100, and 1:100 proteinase inhibitor cocktail) and rotated for 30 min at 4 °C. Nuclei were collected by centrifugation at 2300×*g* for 5 min at 4 °C and rinsed once in 100 μl of 1 × NEBuffer DpnII buffer. Nuclei were resuspended in 120 μl of 0.5% SDS and incubated at 62 °C for 10 min. Nuclei were immediately incubated on ice for 5 min and followed by a 30-min incubation at 37 °C after adding 350 μl of ddH_2_O and 60 μl of 10% Triton X-100 to sequester SDS. Nuclei were mixed with 60 μl of 10 × NEBuffer DpnII buffer and digested using 500 U of DpnII (NEB) on a rotator at 37 °C overnight. DpnII digestion was quenched by incubation at 62 °C for 20 min. Digested nuclei were diluted with 2.4 ml of 1.25 × T4 ligation buffer (300 μl of 10 × NEB T4 ligase buffer, 240 μl of 10% Triton X-100, 1.845 ml of ddH_2_O, freshly added 1:200 proteinase inhibitor cocktail). Nuclei were then ligated by adding 15 μl of NEB T4 DNA ligase (final concentration 30 Weiss U/ml) with rotation overnight at 16 °C. Chromatin was collected by centrifugation at 2300×*g* for 5 min at 4 °C, resuspended in 500 μl 0.5% SDS lysis buffer (0.5% SDS, 10 mM EDTA, 50 mM Tris-HCl, pH 8.0), and sonicated to ~ 500-bp average size on the Branson Sonifier 450 ultrasonic processor (10% amplitude, 0.5 s on 1 s off for 30 s). Chromatin fragments were centrifuged at 16,100×*g* for 10 min at 4 °C. Final concentration 300 mM NaCl was added to the supernatant followed by incubation with 50 μl of MyOne Streptavidin T1 Dynabeads (Thermo Fisher Scientific) at 4 °C. After overnight incubation, Dynabeads were washed twice with 1 ml of 2% SDS, twice with 1 ml of RIPA buffer with 0.5 M NaCl, twice with 1 ml of LiCl buffer, and twice with 1 ml of TE buffer. Chromatin was resuspended in SDS elution buffer (1% SDS, 10 mM EDTA, 50 mM Tris-HCl, pH 8.0, 0.2 mg/ml proteinase K) followed by reverse cross-linking and proteinase K digestion at 65 °C overnight. CAPTURE-3C DNA was purified using QIAquick Spin columns (Qiagen), and 5 ng of DNA was processed for library generation using the NEBNext DNA Library Prep Kit (NEB). Libraries were pooled and 38-bp or 75-bp pair-end sequencing was performed on an Illumina NextSeq500 platform using the 75-bp or 150-bp high output kit.

#### CAPTURE-3C-seq data analysis

Raw reads were processed as previously described [18] with modifications for multiplex capture. First, read pairs of replicate experiments were merged and mapped separately to human (hg19) or mouse (mm10) genome assembly. Unmapped reads were remapped after removing DpnII digestion sites. The mapped reads from both procedures were merged and the reads with low mapping quality were removed. Then the uniquely mapped reads were paired and PCR duplicates were removed. For each sgRNA-targeted region, the preprocessed read pairs were used to define its peak size. If a bait region (enhancer or promoter) contained multiple sgRNAs, their peaks were merged as a single peak for the bait region. For each peak region, the read pairs with both ends within the same peak region were considered as self-ligations and discarded for the downstream analysis. The read pairs with only one end within the peak region were considered as PETs for analyzing long-distance interactions. For each bait region, all chromosomes were binned as the size of its peak size. The statistical significance of the identified intra- and inter-chromosomal PETs were tested as previously described [18]. If a SE region had several enhancers, all the tested PETs were merged as the PETs of this SE. To define the significance of interactions between two bait regions, we implemented a modified Bayesian model [18]. Specifically, given two bait regions *i* and *j* with sizes of *L*(*i*) and *L*(*j*), respectively, we first constructed the random background by randomly sampling two chromosomal regions with the same region sizes of *L*(*i*) and *L*(*j*), and with the same chromosomal distance between them. The random paired samplings were performed 10,000 times by avoiding the overlapping of any bait regions. Then the random PETs of each random paired sampling were extracted and fitted as a negative binomial distribution. Suppose the observed PET number of bait regions *i* and *j* is *x*(*i*, *j*), we can calculate *P* values of random PET *x* less (more) than *x*(*i*, *j*). We then calculated the Bayes factor (BF) to compare the hypothesis H_0_ that random interactions are less than observed interactions against the alternative hypothesis H_1_, representing the complementary case. The BF is defined as $\mathrm{BF}=\frac{\Pr \left(\mathrm{X}<x\left(i,j\right)\right)}{\Pr \left(\mathrm{X}\ge x\left(\mathrm{i},\mathrm{j}\right)\right)}\bullet \frac{\Pr \left({\mathrm{H}}_1\right)}{\Pr \left({\mathrm{H}}_0\right)}$ where $\frac{\Pr \left({\mathrm{H}}_1\right)}{\Pr \left({\mathrm{H}}_0\right)}$ was assigned as 0.001 for controlling false discovery rate (FDR). The BF value larger than 20 was considered as significant interactions between bait regions *i* and *j.*

#### Comparison of chromatin interactions from multiple 3C-based methods

We compared the CAPTURE-3C-seq data with existing datasets for five DHS regions at the β-globin LCR region. The datasets were obtained from NCBI GEO database with accession numbers GSM970213 and GSM970216 for RNAPII and CTCF ChIA-PET; GSM2037371 for UMI-4C; GSM970500 for 5C; GSM2705043, GSM2705044, and GSM27050435 for H3K27ac HiChIP (merged); GSM1370434 and GSM1370436 for DNase Hi-C (merged); and GSM1551618 for in situ Hi-C (Additional file 3: Table S2). For each datatype, the raw reads were processed with the same procedure and parameters as in the CAPTRE-3C-seq data analysis. We then extracted the unique read pairs (PETs) with one end in the targeted enhancer regions and calculated the PETs of an enhancer region as PPKM (PETs Per Kilobase of bait region per Million mapped reads) $\mathrm{PPKM}=\frac{\mathrm{PETs}\times {10}^9}{{\mathrm{Enhance}}_{\mathrm{Size}}\times \mathrm{TotalPairs}}$.

#### Identification of hub enhancers and hierarchical SEs by H-score

To determine the hierarchical structure of SEs, we calculated the relative PET enrichment of enhancers. First, we counted the PET number for enhancer *i* within a SE *j,* denoted as *EN*_*j*_(*i*). The H-score is calculated as ${\mathrm{H}}_j(i)=\frac{EN_j(i)}{\mathrm{L}(i)\times \frac{1}{\mathrm{s}}\sum \limits_1^{\mathrm{s}}\mathrm{EN}(i)}$, where *L*(*i*) is the peak size of enhancer *i* and *s* is the total number of enhancers within the SE *j*. Here, the H-score of an enhancer is defined as mean normalized score, indicating the relative enrichment of interactions among all enhancers within a SE. We then fitted the H-scores of all enhancers as a gamma distribution, and the enhancers with significant H-scores (*P* < 0.05) were referred to as hub enhancers, whereas the other enhancers as non-hub enhancers. If a SE contains at least one hub enhancer, it is categorized as a hierarchical SE, otherwise as a non-hierarchical SE (Additional file 1: Figure S2b). To determine the distance between SEs and their associated gene targets, the genes with significant interactions between their promoters and constituent enhancers within the SEs were extracted using the GENCODE gene annotation V19. For each gene, the distance between its transcription start site (TSS) and interacting enhancers was calculated. If there are multiple enhancers interacting with one gene, the enhancer with the strongest interacting strength was selected for the calculation. Then two distance sets of hierarchical and non-hierarchical SEs were grouped and tested.

#### Analysis of promoter-centric chromatin interactions

The CAPTURE-3C-seq data of promoter-centric chromatin interactions in undifferentiated (0 h) and differentiated (2, 6, 12, and 24 h) G1ER cells were processed as described above. We used the peak sizes of the captured 22 activated promoters and 20 repressed promoters as the anchor regions to identify different types of interactions including enhancer-promoter (E-P) and other interactions. We first annotated enhancers as the genomic regions overlapped with ATAC-seq and H3K27ac ChIP-seq peaks. Then the long-range DNA interactions between an anchor promoter region and its neighboring enhancers within 200 kb are defined as E-P interactions. The rest of intra-chromosomal interactions except E-P interactions were defined as other interactions. The detailed information about the captured promoter regions and the interacting enhancers is shown in Additional file 8: Table S7. To quantify the dynamic kinetics of different types of signals including RNA-seq, ATAC-seq, ChIP-seq, and CAPTURE-3C-seq, we normalized each signal by its own sequencing depth across all time points. Specifically, for RNA-seq, we calculated gene expression as FPKM (Fragments Per Kilobase of transcript per Million mapped reads). For ATAC-seq and ChIP-seq, we calculated FPKM values of the captured promoter region. For CAPTURE-3C-seq, we calculated PPKM (PETs Per Kilobase of bait region per Million mapped reads) for all interactions, E-P interactions, and other interactions. We then generated the normalized signals by the mean of all five time points for each data type (RNA-seq, ATAC-seq, ChIP-seq, and CAPTURE-3C-seq). The resulting normalized FPKM (NFPKM) or normalized PPKM (NPPKM), which indicates the relative signals across all time points for any given data type, was used to compare between different conditions or samples. The enriched TF motifs were identified by HOMER [65] using G+C content controlled genome-wide random background.

### Quantification and statistical analysis

Statistical details including *N*, mean, and statistical significance values are indicated in the text, figure legends, or “Methods” section. Error bars in the experiments represent standard error of the mean (SEM) or standard deviation (SD) from either independent experiments or samples. All statistical analyses were performed using GraphPad Prism, and the detailed information about statistical methods is specified in the figure legends or “Methods” section.

## Supplementary information

**Additional file 1: **Supplementary **Figure S1-S10**.

**Additional file 2:Table S1.** Sequences of sgRNAs and primers.

**Additional file 3:Table S2.** List of genomic datasets. The name, data type, cell type, GEO accession number and citation are shown.

**Additional file 4:Table S3.** List of identified locus-specific chromatin interactions at β-globin LCR. The list of locus-specific inter- and intra-chromosomal DNA interactions identified by multiplexed CAPTURE-3C-seq analysis of β-globin LCR is shown. Data processing and statistical analyses were performed as described in Methods. Results compiled from two or three independent CAPTURE-3C-seq experiments are shown.

**Additional file 5:Table S4.** List of captured hierarchical and non-hierarchical SEs. Hi-Hub, hub enhancer within the hierarchical SE; Hi-NonHub, non-hub enhancer within the hierarchical SE; NonHi-NonHub, non-hierarchical SE.

**Additional file 6:Table S5.** List of identified locus-specific chromatin interactions at the captured erythroid SEs. Results compiled from two or three independent CAPTURE-3C-seq experiments are shown.

**Additional file 7:Table S6.** List of identified promoter-centric chromatin interactions in G1ER cells. The lists of locus-specific interactions identified by multiplexed CAPTURE-3C-seq analysis of 22 activated and 20 repressed promoters are shown. Results compiled from two or three independent CAPTURE-3C-seq experiments are shown.

**Additional file 8:Table S7.** List of candidate enhancers interacting with captured promoters in G1ER cells. The chromosome coordinates of the candidate enhancers interacting with the captured promoters are shown.

**Additional file 9:** Review history.

## Data Availability

All raw and processed ATAC-seq, ChIP-seq, RNA-seq, CAPTURE-ChIP-seq, and CAPTURE-3C-seq data are available in the Gene Expression Omnibus (GEO): GSE139117 (https://www.ncbi.nlm.nih.gov/geo/query/acc.cgi?acc=GSE139117) [66]. The computer code for sgRNA design is available from GitHub (https://github.com/Yuannyu/CAPTURE2.0_sgRNA_design) [67]. The computer code for CAPTURE-3C-seq data processing and analysis is available from GitHub (https://github.com/ChenYong-RU/MAXIM) [68]. Other codes are available from the corresponding author on request.
