# Supplementary material for: Multiplexed capture of spatial configuration and temporal dynamics of locus-specific 3D chromatin by biotinylated dCas9
Source: Genome Biol. 2020 Mar 5;21:59. doi: 10.1186/s13059-020-01973-w (PMC7059722; doi:10.1186/s13059-020-01973-w)
Supplement: Supplementary file 1 — Additional file 1: Supplementary Figure S1-S10. [file 13059_2020_1973_MOESM1_ESM.pdf]

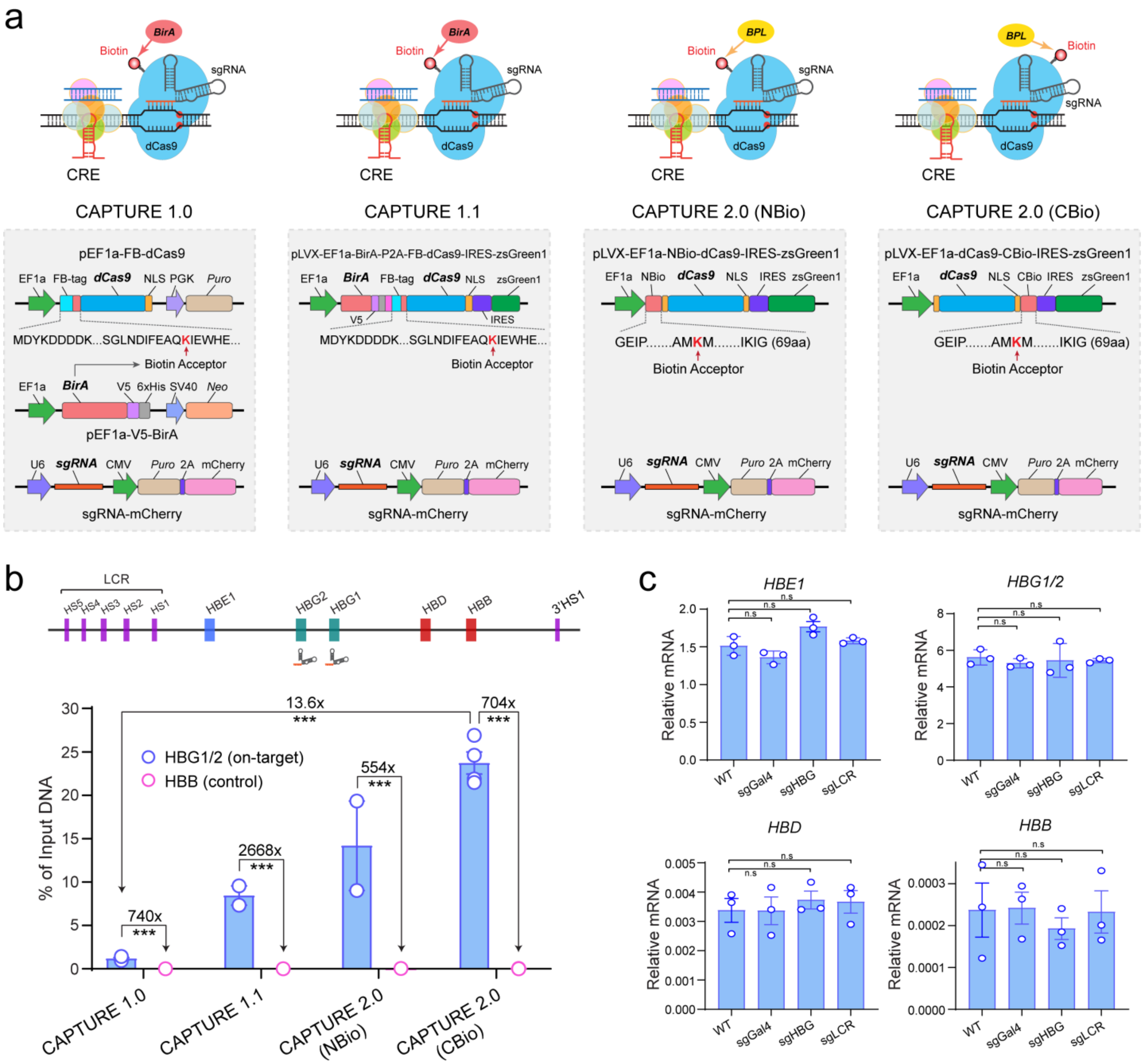

Figure S1. Development of the evolving CRISPR CAPTURE systems

(a) Schematic of vector design for the evolving CAPTURE systems.

(b) Comparisons of capture efficiency and on-target enrichment of various CAPTURE systems. CAPTURE-ChIP-qPCR was performed in K562 cells co-expressing *HBG1/2*-specific sgRNA and biotinylated dCas9 protein. The ChIP signal as % of input DNA using PCR primers for the sgRNA-targeted *HBG1/2* promoter and the non-targeted *HBB* promoter (control). The fold enrichment of ChIP signals is shown. Results are mean ± SEM of N = 3 or 4 experiments and analyzed by a two-sided t-test. ***$P < 0.001$.

(c) Expression of human β-globin genes (*HBE1*, *HBG1/2*, *HBD* and *HBB*) were unaffected by co-expression of dCas9 with target-specific (sgHBG or sgLCR) or non-targeting sgRNAs relative to wild-type (WT) K562 cells. The expression of β-globin mRNAs was analyzed by qRT-PCR. Results are mean ± SEM of N = 3 independent experiments.

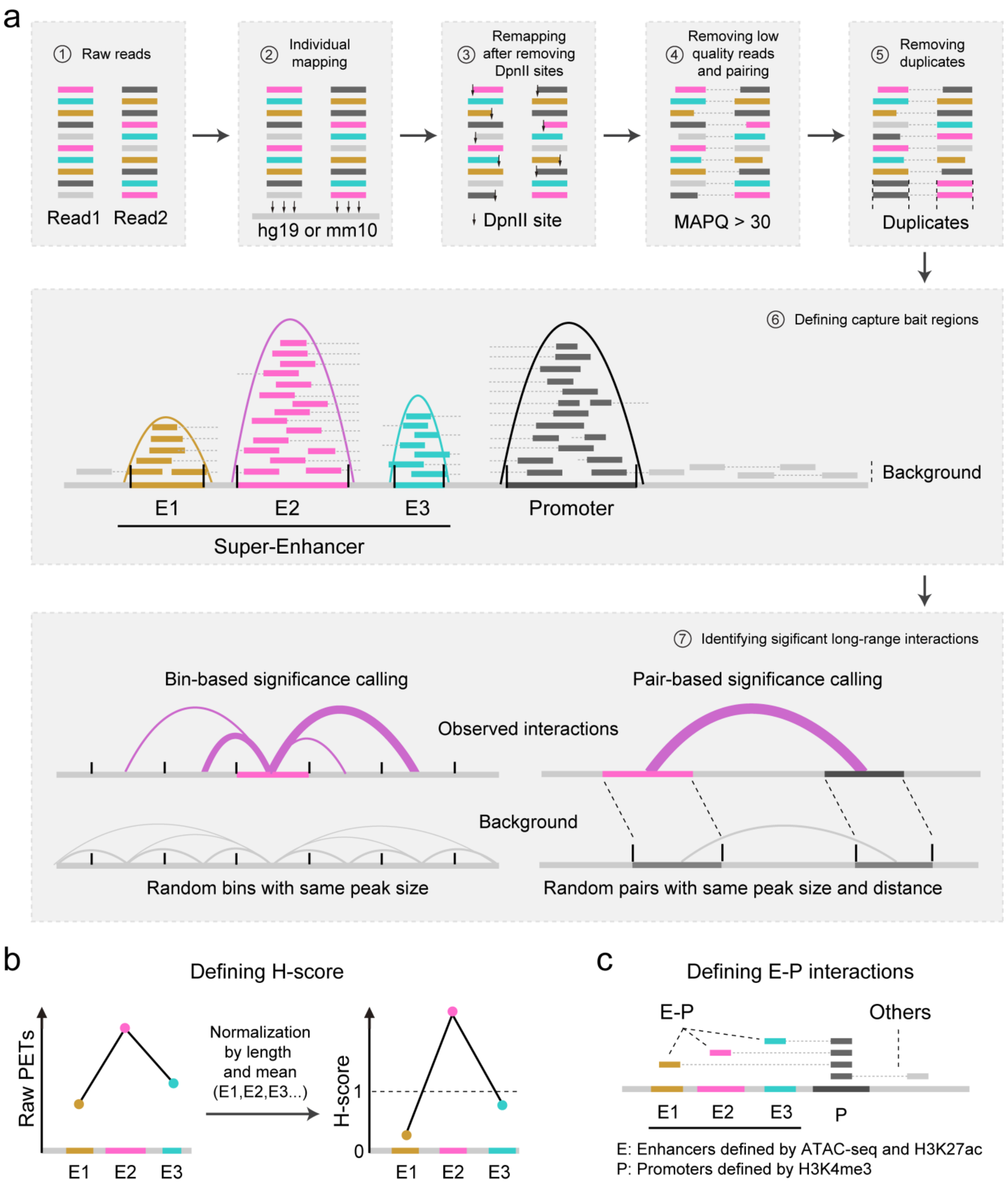

Figure S2. A computational workflow for CAPTURE-3C-seq data analysis

(a) Data processing and statistical analysis pipeline for CAPTURE-3C-seq.
(b) Schematic of the computational metric for the identification of hub enhancers by H-score.
(c) Schematic of the workflow to identify E-P interactions and other interactions by multiplexed capture.

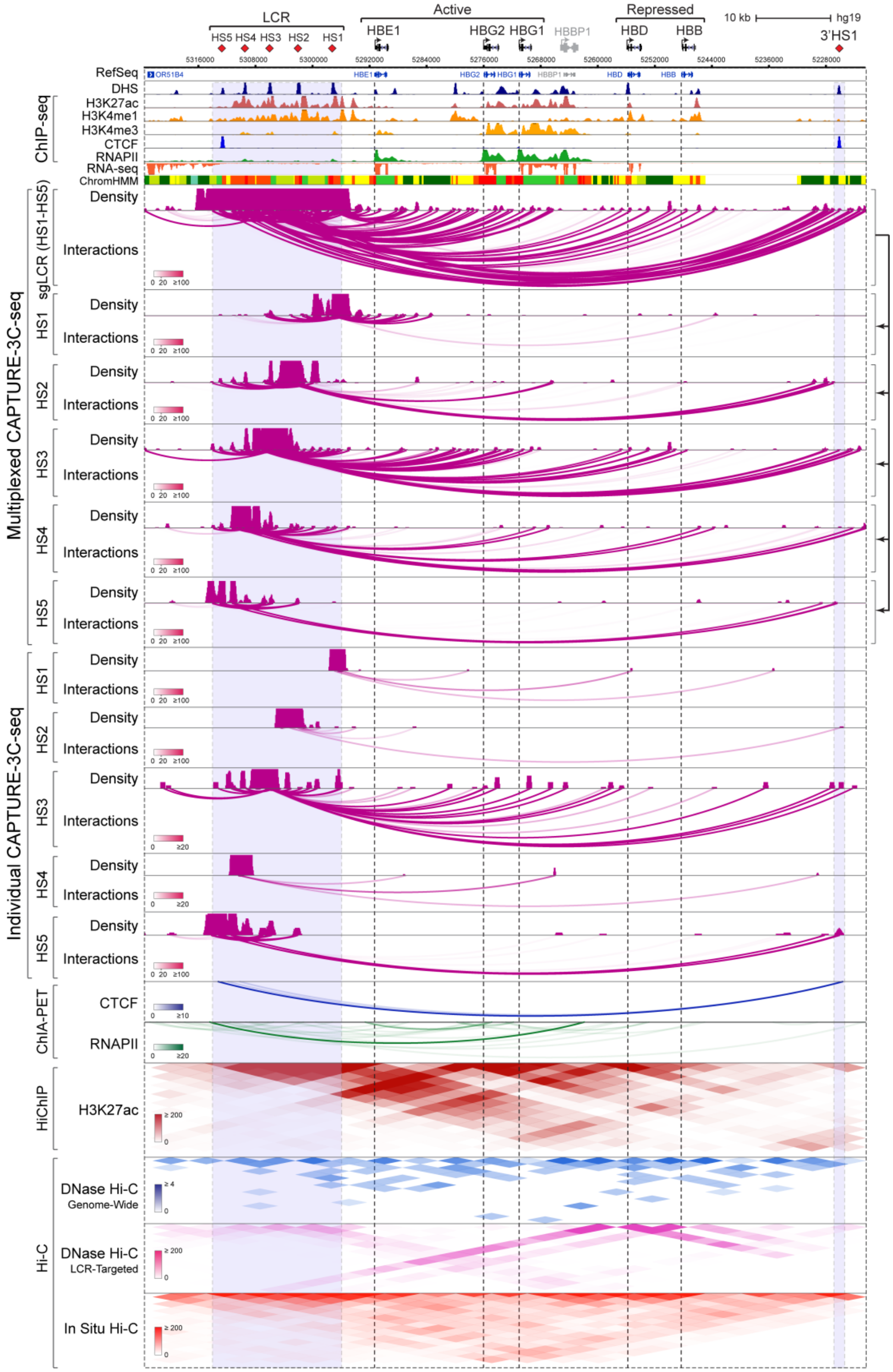

Figure S3. Analysis of locus-specific chromatin interactions by multiplexed and individual CAPTURE experiments

Browser view of the long-range DNA interaction profiles at LCR enhancers identified by multiplexed or individual CAPTURE analyses is shown (chr11:5,222,424-5,323,623; hg19). Contact profiles compiled from two or three CAPTURE-3C-seq experiments including the density map and interactions are shown for LCR and each HS enhancer (HS1 to HS5). DHS, ChIP-seq, RNA-seq, ChromHMM, ChIA-PET (CTCF and RNAPII) [1, 2], HiChIP (H3K27ac) [3], DNase Hi-C [4], and *in situ* Hi-C [5] data are shown for comparison.

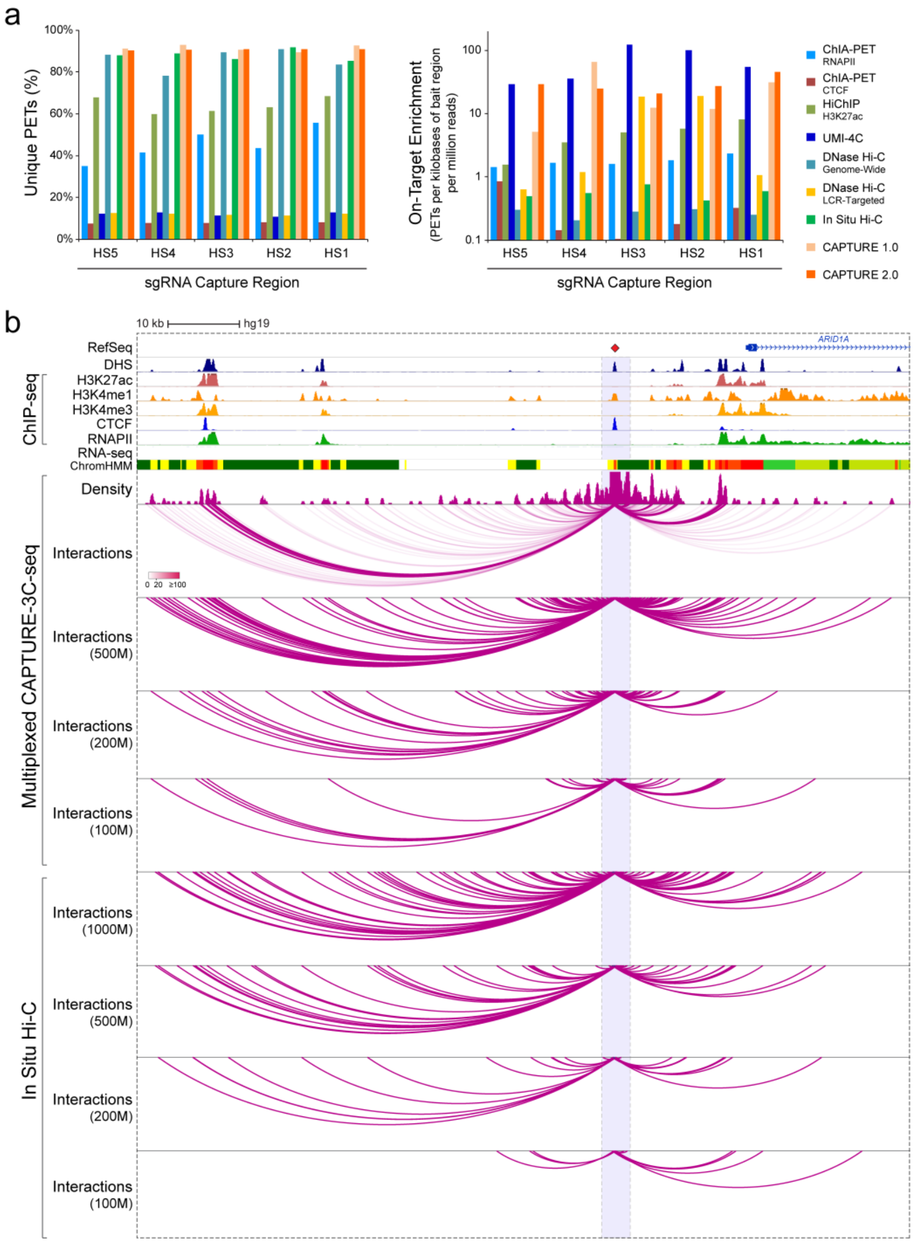

Figure S4. Comparions between CAPTURE and other 3C-based methods

(a) Comparisons between ChIA-PET (RNAPII and CTCF) [1, 2], HiChIP (H3K27ac) [3], DNase Hi-C [4] (genome-wide or LCR-targeted), *in situ* Hi-C [5], UMI-4C [6], and CAPTURE-3C-seq (CAPTURE1.0 and CAPTURE2.0) in K562 cells are shown. Comparing with ChIA-PET, CAPTURE-3C-seq shows significantly higher % of unique PETs and on-target enrichment as measured by the number of PET interactions per kb of bait region per million mapped reads. The unique PETs were defined as pair-end sequence tags with distinct genomic locations at one or both sides of the pair-end reads. Comparing with Hi-C, CAPTURE-3C-seq shows comparable or slightly higher % of unique PETs but significantly higher on-target enrichment. Comparing with UMI-4C, CAPTURE-3C-seq displayed higher % of unique PETs but comparable or slightly lower on-target enrichment. Comparing with H3K27ac HiChIP, CAPTURE-3C-seq displayed higher % of unique PETs but comparable or slightly higher on-target enrichment.

(b) Browser view of CRE-mediated long-range interactions (chr1:26,937,310-27,045,510; hg19) identified by multiplexed CAPTURE-3C-seq and *in situ* Hi-C. Contact profiles including the density map and interactions for the dCas9-captured LCR region or the resolved individual enhancers are shown. Down-sampling of multiplexed CAPTURE-3C-seq or Hi-C using 100M, 200M, 500M and 1000M reads is shown for comparison.

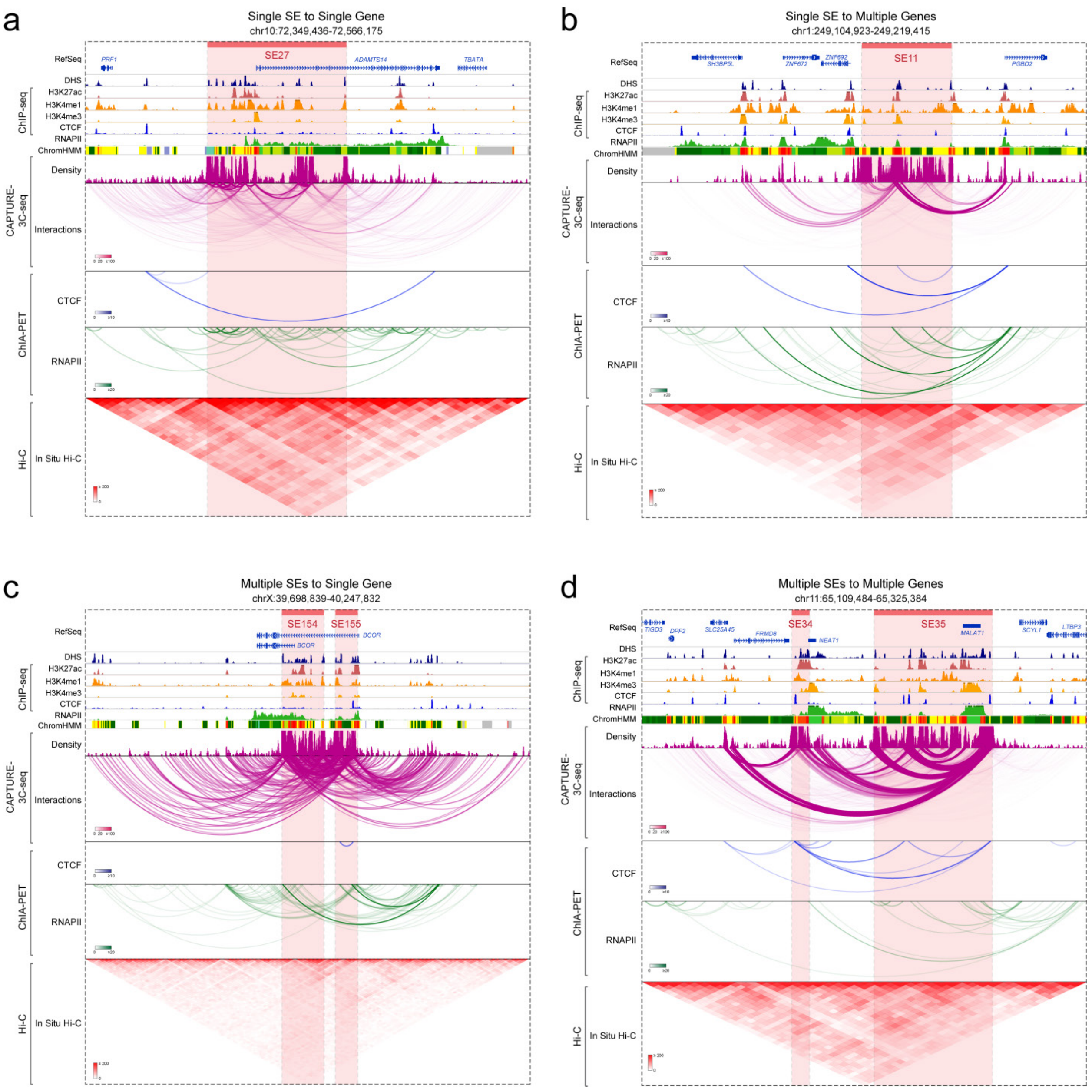

Figure S5. Representative loci for super-enhancer and target gene interactions

(a) A representative locus is shown for single SE to single gene interactions (SE27). Contact profiles including the density map and interactions for the dCas9-captured SE region (*red* bar) are shown. The statistical significance of interactions was determined by the Bayes Factor (BF) and indicated by the color scale bars. DHS, ChIP-seq, ChromHMM, ChIA-PET (CTCF and RNAPII) and *in situ* Hi-C data are shown for comparison.

(b) A representative locus is shown for single SE to multiple genes (SE11).

(c) A representative locus is shown for multiple SEs to single gene (SE154 and SE155).

(d) A representative locus is shown for multiple SEs to multiple genes (SE34 and SE35).

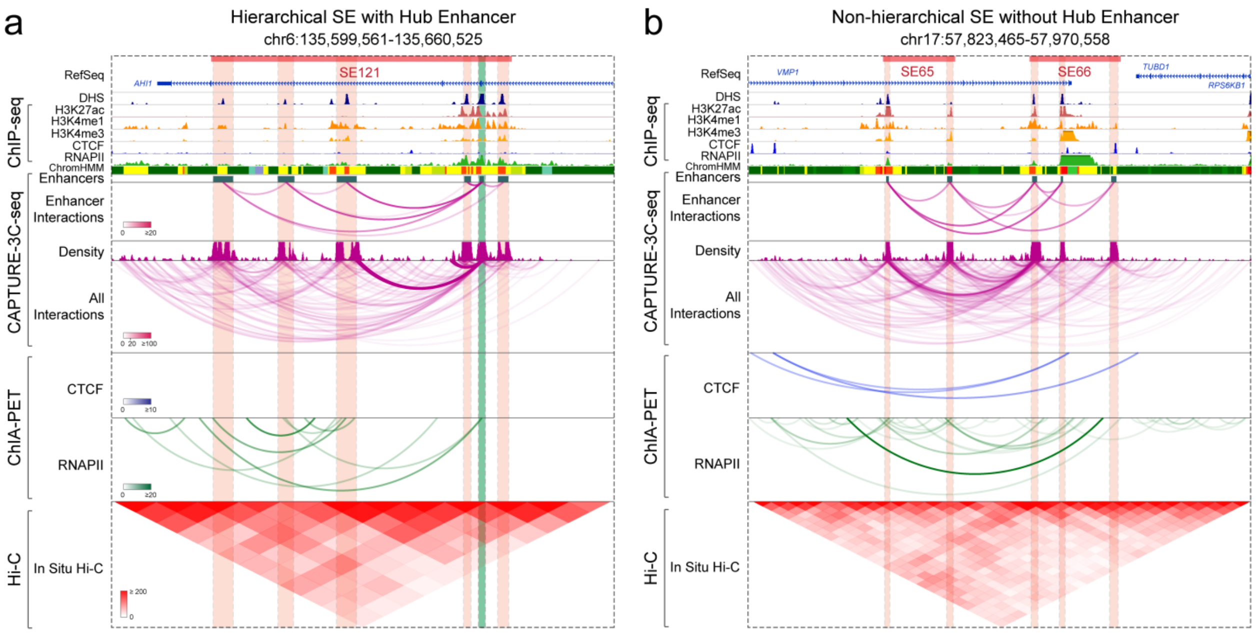

c

Hierarchical SEs	
Motif Name	*P* value
JUN	1.00E-17
FOSL2	1.00E-13
ATF3	1.00E-13
BATF	1.00E-13
AP-1	1.00E-09
GATA2	1.00E-08
GATA1	1.00E-08
NRF2	1.00E-07
CTCF	1.00E-07
GATA1:TAL1	1.00E-05

Non-Hierarchical SEs	
Motif Name	*P* value
JUN	1.00E-18
FOSL2	1.00E-18
GATA2	1.00E-16
GATA1	1.00E-15
GATA1:TAL1	1.00E-12
BATF	1.00E-12
AP-1	1.00E-10
ATF3	1.00E-10
BACH1	1.00E-09
NRF2	1.00E-08

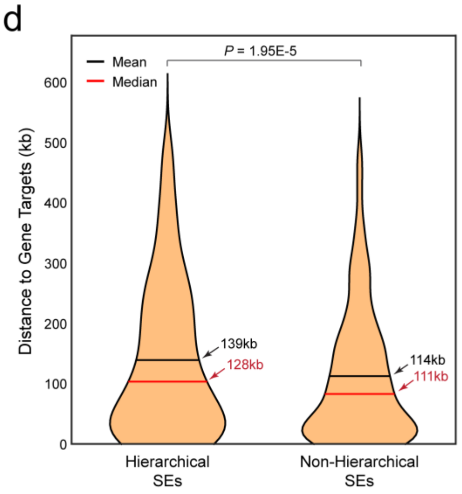

Figure S6. Representative loci for hierarchical and non-hierarchical super-enhancers

(a) A representative locus is shown for a hierarchical SE containing the hub enhancer (SE121). Contact profiles including the density map, interactions between enhancers and all interactions for the dCas9-captured SE region (*red* bar) are shown. The identified hub and non-hub enhancers are depicted by *green* (hub) and *red* (non-hub) lines, respectively.

(b) A representative locus is shown for a non-hierarchical SE without hub enhancer (SE65 and SE66).

(c) Top enriched TF motifs associated with hierarchical and non-hierarchical SEs. The name and *P* value of enrichment for each motif are shown.

(d) Violin plots are shown for the distance between hierarchical or non-hierarchical SEs and their gene targets. The mean and median distances are shown and indicated by *black* and *red* lines, respectively. *P* value was calculated by the Student's *t*-test.

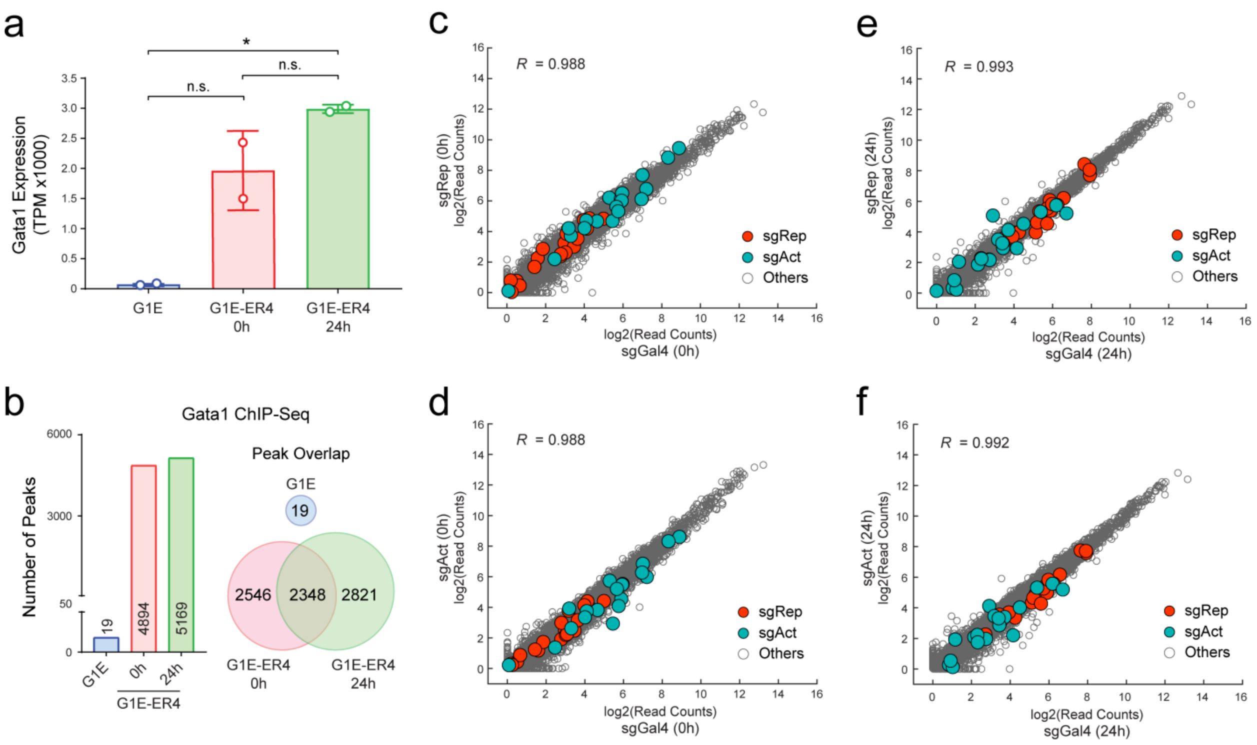

Figure S7. Transcriptomic analyses of G1ER erythroid differentiation

(a) Expression of *Gata1* mRNA in G1E, undifferentiated (0h) and differentiated (24h) G1ER cells. Results are mean ± SD of the normalized transcripts per million (TPM) values (N = 2 RNA-seq experiments), and analyzed by Welch's ANOVA followed by Dunnett's T3 multiple comparisons. *P < 0.05, n.s. not significant.

(b) The numbers of GATA1 ChIP-seq in G1E, undifferentiated (0h) and differentiated (24h) G1ER cells (left). Venn diagram is shown for the overlap analysis of GATA1 ChIP-seq peaks in G1E, undifferentiated (0h) and differentiated (24h) G1ER cells (right).

(c) Differential gene expression analysis was performed using RNA-seq in undifferentiated (0h) G1ER cells expressing dCas9-CBio with sgRep or non-targeting sgGal4. The captured activated or repressed promoter-associated genes are indicated by *green* and *red*, respectively. Pearson correlation coefficient (R) value is calculated for each comparison (N = 3 RNA-seq experiments).

(d) Differential gene expression analysis in undifferentiated (0h) G1ER cells expressing dCas9-CBio with sgAct or sgGal4.

(e) Differential gene expression analysis in differentiated (24h) G1ER cells expressing dCas9-CBio with sgRep or sgGal4.

(f) Differential gene expression analysis in differentiated (24h) G1ER cells expressing dCas9-CBio with sgAct or sgGal4.

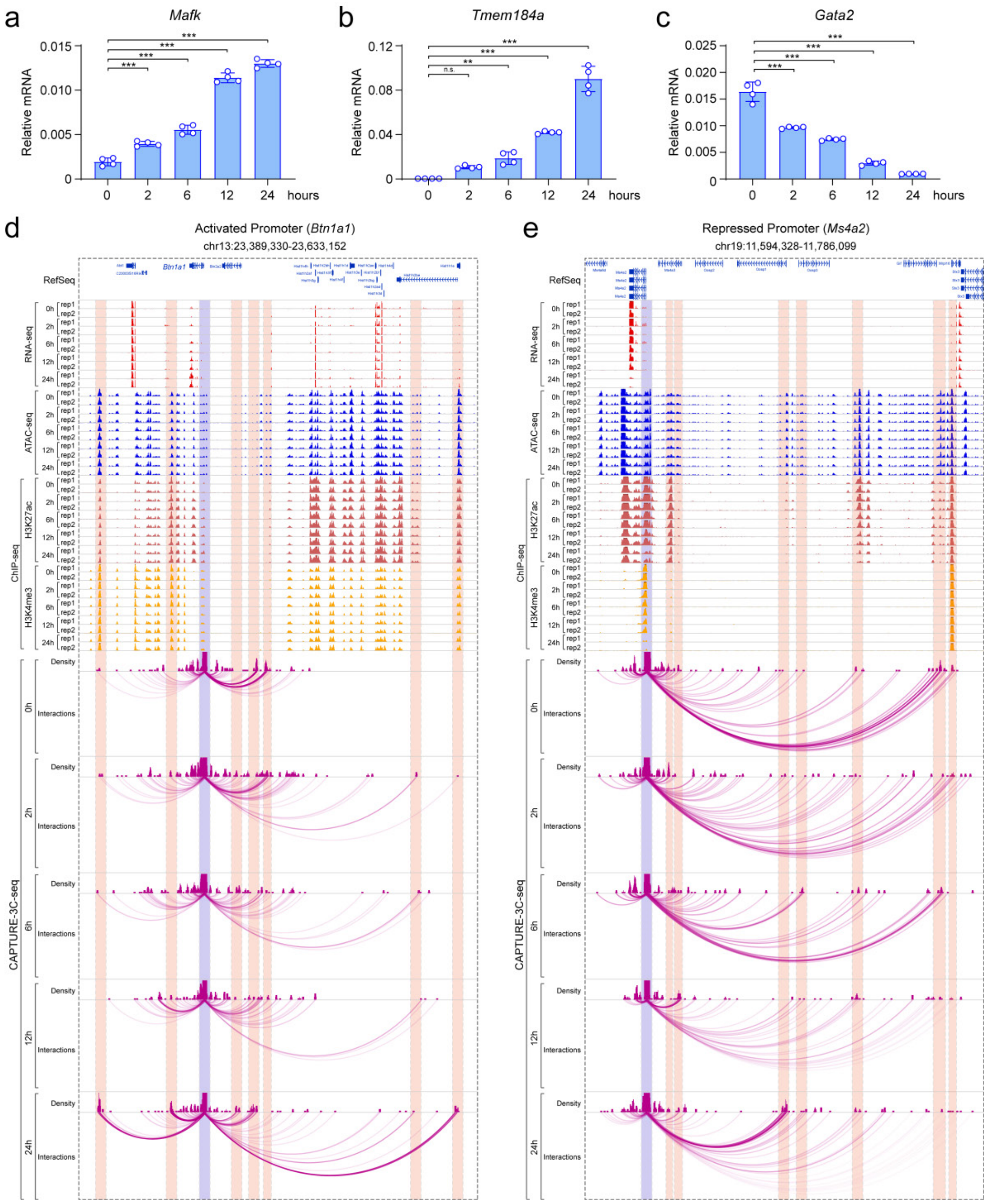

Figure S8. Representative loci for captured promoter-centric interactions during erythroid differentiation

(a) Expression of *Mafk* mRNA during G1ER differentiation (0 to 24h). The mRNA expression relative to Gapdh was determined by qRT-PCR. Results are shown as mean ± SEM (N = 4 independent experiments), and analyzed by one-way ANOVA for multiple comparisons. **P < 0.01, ***P < 0.001, n.s. not significant.

(b) Expression of *Tmem184a* mRNA during G1ER differentiation (0 to 24h).

(c) Expression of *Gata2* mRNA during G1ER differentiation upon GATA1 activation (0 to 24h).

(d) A representative locus is shown for the activated promoter (*Btn1a1*)-mediated chromatin interactions during erythroid differentiation. Contact profiles including the density map and interactions for the dCas9-captured target region (*blue* bar) and the interacting CREs (*red* bar) are shown. The statistical significance of interactions was determined by the Bayes Factor (BF) and indicated by the color scale bars. Two independent replicate experiments (rep1 and rep2) are shown for RNA-seq, ATAC-seq and ChIP-seq in G1ER cells at varying time points of GATA1-induced erythroid differentiation (0, 2, 6, 12 and 24h).

(e) A representative locus is shown for the repressed promoter (*Ms4a2*)-mediated chromatin interactions during erythroid differentiation.

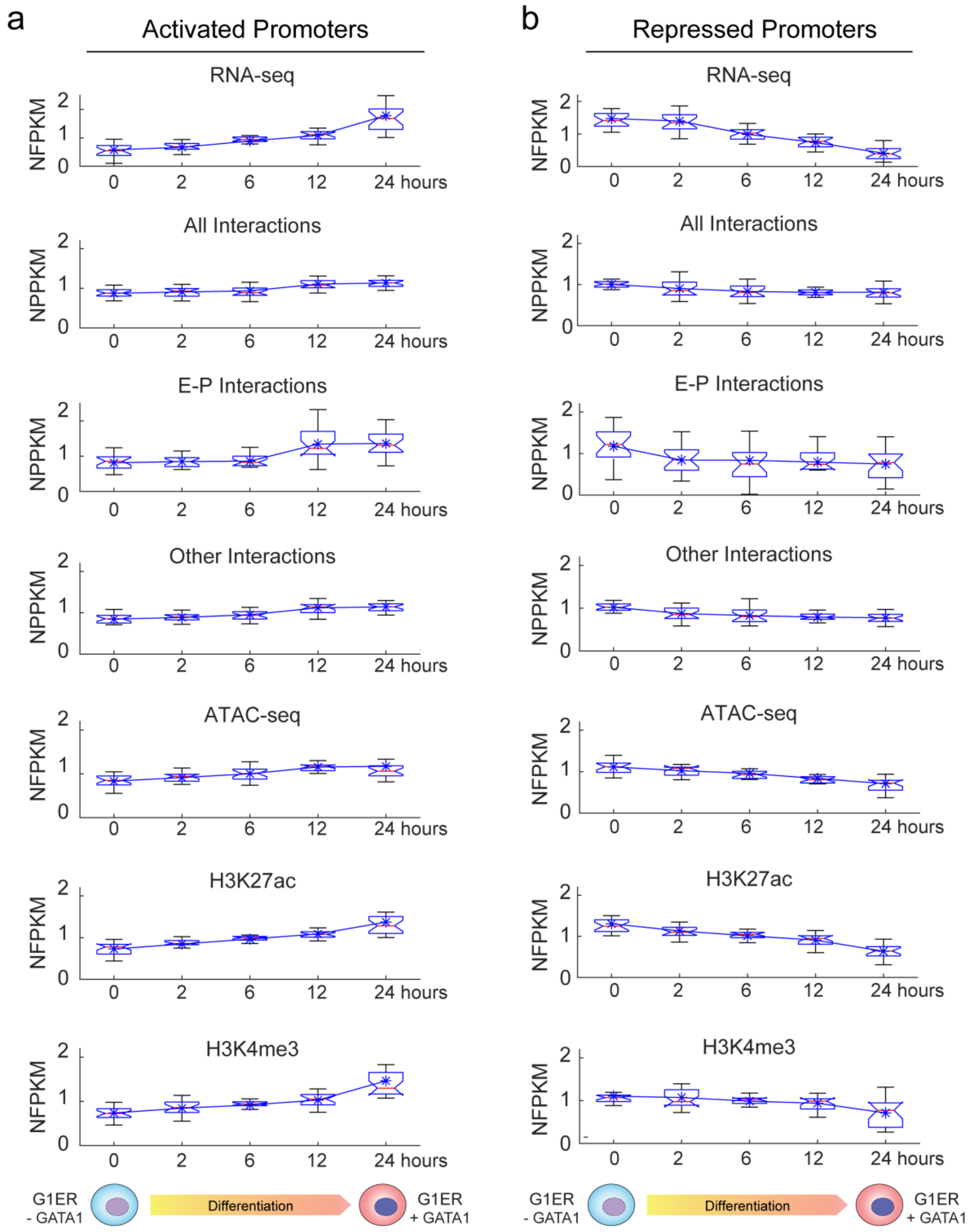

Figure S9. Correlation analysis of promoter-centric interactions, gene expression and epigenetic landscapes

(a) The changes in gene expression by RNA-seq, chromatin interactions (all, E-P and other interactions) by CAPTURE-3C-seq, chromatin accessibility by ATAC-seq, and histone marks (H3K27ac and H4K4me3) by ChIP-seq associated with dCas9-captured activated promoters at various time points of G1ER differentiation (0 to 24h). The x-axis denotes the time points of undifferentiated (0h) or differentiated (2, 6, 12 and 24h) G1ER cells. The y-axis denotes the normalized signals calculated by the mean normalized gene FPKM (NFPKM for RNA-seq), reads counts (NFPKM for ATAC-seq and ChIP-seq), or PETs (NPPKM for CAPTURE-3C-seq) per kb of captured genomic region per million mapped reads (see Methods), and shown as mean ± SEM (*N* = 22 activated promoters).

(b) The changes in gene expression, chromatin interactions, chromatin accessibility, and histone marks associated with dCas9-captured repressed promoters during G1ER differentiation (0 to 24h). The y-axis denotes the normalized signals and shown as mean ± SEM (*N* = 20 repressed promoters).

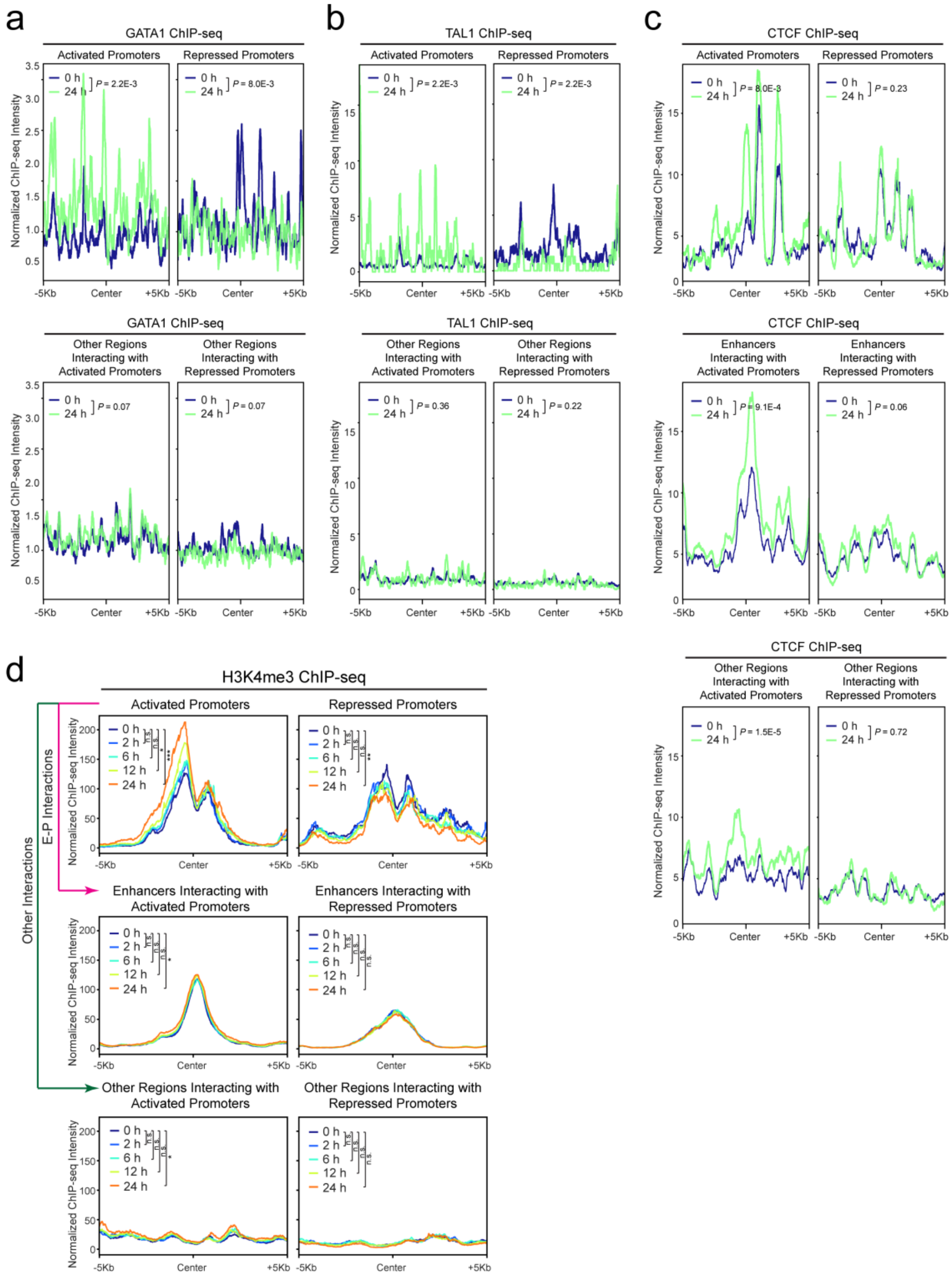

Figure S10. Chromatin occupancy of TFs at captured promoters and interacting regions

(a) Chromatin occupancy of GATA1 at the captured activated or repressed promoters, and other regions interacting with activated or repressed promoters in undifferentiated (0h) and differentiated (24h) G1ER cells. *P* values were calculated using the two-sample Kolmogorov-Smirnov (K-S) test.

(b) Chromatin occupancy of TAL1 at the captured activated or repressed promoters, and other regions interacting with activated or repressed promoters in undifferentiated (0h) and differentiated (24h) G1ER cells.

(c) Chromatin occupancy of CTCF at the captured activated or repressed promoters, enhancers and other regions interacting with activated or repressed promoters in undifferentiated (0h) and differentiated (24h) G1ER cells.

(d) Spatial distribution of H3K4me3 ChIP-seq signals at the captured activated or repressed promoters, enhancers and other regions interacting with activated or repressed promoters in undifferentiated and differentiated G1ER cells.

References

1. Consortium TEP: An integrated encyclopedia of DNA elements in the human genome. *Nature* 2012, 489:57-74.
2. Li G, Ruan X, Auerbach RK, Sandhu KS, Zheng M, Wang P, Poh HM, Goh Y, Lim J, Zhang J, et al: Extensive promoter-centered chromatin interactions provide a topological basis for transcription regulation. *Cell* 2012, 148:84-98.
3. Mumbach MR, Satpathy AT, Boyle EA, Dai C, Gowen BG, Cho SW, Nguyen ML, Rubin AJ, Granja JM, Kazane KR, et al: Enhancer connectome in primary human cells identifies target genes of disease-associated DNA elements. *Nat Genet* 2017, 49:1602-1612.
4. Ma W, Ay F, Lee C, Gulsoy G, Deng X, Cook S, Hesson J, Cavanaugh C, Ware CB, Krumm A, et al: Fine-scale chromatin interaction maps reveal the cis-regulatory landscape of human lincRNA genes. *Nat Methods* 2015, 12:71-78.
5. Rao SS, Huntley MH, Durand NC, Stamenova EK, Bochkov ID, Robinson JT, Sanborn AL, Machol I, Omer AD, Lander ES, Aiden EL: A 3D map of the human genome at kilobase resolution reveals principles of chromatin looping. *Cell* 2014, 159:1665-1680.
6. Schwartzman O, Mukamel Z, Oded-Elkayam N, Olivares-Chauvet P, Lubling Y, Landan G, Izraeli S, Tanay A: UMI-4C for quantitative and targeted chromosomal contact profiling. *Nat Methods* 2016, 13:685-691.
