# Supplementary material for: Multiplexed capture of spatial configuration and temporal dynamics of locus-specific 3D chromatin by biotinylated dCas9
Source: Genome Biol. 2020 Mar 5;21:59. doi: 10.1186/s13059-020-01973-w (PMC7059722; doi:10.1186/s13059-020-01973-w)
Supplement: Supplementary file 9 — Additional file 9: Review history. [file 13059_2020_1973_MOESM9_ESM.docx]

Review History

**First round of review**

**Reviewer 1**

**Were you able to assess all statistics in the manuscript, including the appropriateness of statistical tests used?**

Yes: Statistics used were clearly explained in the Methods.

**Were you able to directly test the methods?**

No

**Comments to author:**

In this paper, authors introduce a new version of CRISPR Affinity Purification in situ of Regulatory Elements, or CAPTURE2.0, to isolate endogenous regulatory element protein complexes and determine 3D long-range chromatin interactions at chosen elements. Several modifications to the original CAPTURE are introduced. Briefly, by adopting a BioTAP-tag, which can be recognized by endogenously expressed biotin protein ligases and placing the tag at the C-terminal of dCAS9, capture efficiency was greatly improved while on-target enrichment was largely comparable. Authors then carry out CAPTURE2.0 3-C-seq with multiplexed sgRNAs simultaneously targeting the DNase hypersensitive sites 1-5 of the beta-globin LCR. Resolving the multiplex captured LCR-mediated interactions to individual hypersensitive sites computationally provided very similar results to previous published work using CAPTURE1.0 with individual sgRNAs in multiple experiments. This comparison provides a validation of multiplexed CAPTURE2.0 with somewhat higher % of unique PETS and on-target enrichment compared to CAPTURE1.0.

Authors then apply the multiplex approach to study the organizational principles underlying super enhancers (SEs). Here they multiplexed 1,870 sgRNAs to target individual enhancers within the top 157 SEs in K562 cells obtaining a high capture efficiency analysis of hundreds of independent regulatory elements and a higher resolution of interactions than CTCF or Pol II ChIA-PET or HiC. Importantly, authors use the new protocol to examine the temporal dynamics of long-range enhancer interactions during differentiation of erythroid G1E cells. Using pooled sgRNAs for promoter capture and examining the most repressed or activated genes during differentiation, they found gain/loss of enhancer-promoter (E-P) interactions correlated with up/down transcription changes and preceded significant changes in epigenetic modifications and chromatin accessibility. This result supports the model that E-P loop gain/loss causally underlies gene activation/repression. Finally, authors analyze transcription factor motifs and find erythroid master regulators at promoters involved in enhancer interactions and significant gain/loss of occupancy in concert with gain/loss of interactions during differentiation, indicating the importance of the transcription factors to the loop changes. This supports an instructive role for E-P interactions in gene expression in response to master erythroid lineage transcription factors.

Overall, the CAPTURE2.0 approach is a significant improvement over the first version, allowing flexibility to use primary cells and to multiplex sgRNAs to have comparable temporal interaction data from the same experiment. It will be of interest to a broad readership. In addition to the method presentation, authors provide thoughtful interpretation of data with respect to models of gene regulation by enhancers.

Comments:

1. Page 9, lines 12-14: Data in figure 3 is used to support the contention that CTCF/cohesin are essential for hub enhancer-promoter interactions. But figure 5 data support the role of master transcription regulators in E-P loop formation and the CTCF motif is not one of the strong motifs observed at promoters involved in erythroid E-P interactions. Do the figure 5 interactions not include hub enhancers? For the figure 5 E-P loops, are CTCF/master regulators enriched at the enhancers? Authors should clarify the direct contributions of CTCF/cohesin vs master transcription regulators to E-P loops.

2. Page 10, line 46; Page 11, line 39: Please supply more information (for example, number of enhancers) and reference or description for 'annotated enhancers' and indicate in figures by name or location w/respect to TSS for examples used.

3. Page 11, line 42: In figure 5d are E-P interactions really more significantly and positively correlated with expression changes than 'other interactions'? It is difficult for this reader to distinguish the orange and red lines in figure 5a-d—a different color choice would help.

4. Figure 2a: sgRNAs are said to be close to but not overlapping enhancers but in Figure 2a it looks like the sgRNA on the right in within the enhancer.

**Reviewer 2**

**Were you able to assess all statistics in the manuscript, including the appropriateness of statistical tests used?**

Yes: All appropriate.

**Were you able to directly test the methods?**

No

**Comments to author:**

In this manuscript, Liu et al aim to interrogate chromatin organization at cis-regulatory elements (CRE) such as enhancers and promoters at high resolution in a multiplexed manner. To this aim, they utilize an improved version of their previously published dCas9-based CAPTURE method, which utilizes biotinylation of dCas9 at genomic elements followed by streptavidin-based affinity purification and analysis of long-range interactions by 3C based technologies. In this paper, they improve on CAPTURE (CAPTURE 2.0) by incorporating a 75 bp biotinylation targeting sequence (BioTAP) to dCas9 that can be recognized by endogenous biotin-protein ligases (BPLs) in eukaryotic cells, which demonstrates ease of cell line engineering and increase in capture efficiency. Using the improved CAPTURE, they explore multiplexing many guide RNAs to analyze multiple CREs in a single experiment. They demonstrate that they can dissect interactions from each enhancer at the beta globin locus control region in high resolution and identify differences in organization. They further assay individual constituent enhancers at many enhancer clusters i.e. super enhancers (SE), and were able to identify qualitative and quantitative differences in long-range interactions of each CREs. They further assay promoter centered dynamic interactions in a differentiation setting. Through these demonstrations, they make a point of potential of CAPTURE in understanding chromatin organization in regulatory elements in high-resolution without any need of a priori knowledge of protein factors at said elements.

Previously, the same group have developed the CAPTURE-3C-Seq method to analyze long -range interactions from individual regulatory elements. Improving up on their previous method, here they demonstrate the potential of multiplexed CAPTURE on identifying principles of chromatin organization at important regulatory elements. This improved and multiplexed method would be of great interest to the field of chromatin organization and gene regulation.

Comments:

1.     In the paper, CAPTURE-3C-Seq interactions are compared to Hi-C and ChIA-PET interactions. How does CAPTURE compare to recent methods such as Plac-Seq / HiChIP in capturing enhancer/ promoter centric interactions? For the browser views for the enhancer CAPTURE experiments, it would be beneficial to see published H3K27ac ChIA-PET / Plac-Seq / HiChIP interactions. As they also demonstrate in Figure 3, CTCF and cohesin is enriched in a subset of enhancers called hub enhancers whereas most of these enhancers still engage in long-range interactions.

2.     Are the same gRNAs used for the multiplexed vs individual CAPTURE-3C-Seq experiments of the globin locus?

3.     How many of the selected SEs / constituent enhancers are intergenic / intragenic?

4.     Do you see any other differences between hub-enhancer containing vs non-hierarchical super enhancers? Any differences in their distance to the target genes? Differences in motifs for hub vs non-hub enhancers?

5.     Please explain GATA1 transgene expression dynamics upon estradiol treatment in Gata1-null G1ER cells. Prior to differentiation, at time 0 of estradiol treatment, how much Gata1 is expressed that you see enrichment at GATA1-ChIP Seq?

**Authors Response**

**Point-by-point responses to the reviewers’ comments:**

Reviewer #1:

In this paper, authors introduce a new version of CRISPR Affinity Purification in situ of Regulatory Elements, or CAPTURE2.0, to isolate endogenous regulatory element protein complexes and determine 3D long-range chromatin interactions at chosen elements. Several modifications to the original CAPTURE are introduced. Briefly, by adopting a BioTAP-tag, which can be recognized by endogenously expressed biotin protein ligases and placing the tag at the C-terminal of dCAS9, capture efficiency was greatly improved while on-target enrichment was largely comparable. Authors then carry out CAPTURE2.0 3-C-seq with multiplexed sgRNAs simultaneously targeting the DNase hypersensitive sites 1-5 of the beta-globin LCR. Resolving the multiplex captured LCR-mediated interactions to individual hypersensitive sites computationally provided very similar results to previous published work using CAPTURE1.0 with individual sgRNAs in multiple experiments. This comparison provides a validation of multiplexed CAPTURE2.0 with somewhat higher % of unique PETS and on-target enrichment compared to CAPTURE1.0.

Authors then apply the multiplex approach to study the organizational principles underlying super enhancers (SEs). Here they multiplexed 1,870 sgRNAs to target individual enhancers within the top 157 SEs in K562 cells obtaining a high capture efficiency analysis of hundreds of independent regulatory elements and a higher resolution of interactions than CTCF or Pol II ChIA-PET or HiC. Importantly, authors use the new protocol to examine the temporal dynamics of long-range enhancer interactions during differentiation of erythroid G1E cells. Using pooled sgRNAs for promoter capture and examining the most repressed or activated genes during differentiation, they found gain/loss of enhancer-promoter (E-P) interactions correlated with up/down transcription changes and preceded significant changes in epigenetic modifications and chromatin accessibility. This result supports the model that E-P loop gain/loss causally underlies gene activation/repression. Finally, authors analyze transcription factor motifs and find erythroid master regulators at promoters involved in enhancer interactions and significant gain/loss of occupancy in concert with gain/loss of interactions during differentiation, indicating the importance of the transcription factors to the loop changes. This supports an instructive role for E-P interactions in gene expression in response to master erythroid lineage transcription factors.

Overall, the CAPTURE2.0 approach is a significant improvement over the first version, allowing flexibility to use primary cells and to multiplex sgRNAs to have comparable temporal interaction data from the same experiment. It will be of interest to a broad readership. In addition to the method presentation, authors provide thoughtful interpretation of data with respect to models of gene regulation by enhancers.

*We appreciate the positive feedback on our manuscript.*

Comments:

1.	Page 9, lines 12-14: Data in figure 3 is used to support the contention that CTCF/cohesin are essential for hub enhancer-promoter interactions. But figure 5 data support the role of master transcription regulators in E-P loop formation and the CTCF motif is not one of the strong motifs observed at promoters involved in erythroid E-P interactions. Do the figure 5 interactions not include hub enhancers? For the figure 5 E-P loops, are CTCF/master regulators enriched at the enhancers? Authors should clarify the direct contributions of CTCF/cohesin vs master transcription regulators to E-P loops.

*We appreciate these thoughtful comments and have provided clarifications in the revised texts. Specifically, we did not perform the analysis to distinguish hub vs non-hub enhancers in promoter-centric capture in G1ER cells.*

*This analysis would require high-resolution multiplexed capture of super-enhancers (SEs) and their constituent enhancers in G1ER cells as we did in K562 cells (Fig. 3). Instead, the promoter-centric capture focused on the kinetic analysis of a subset of activated and repressed promoters as well as their interacting genomic regions such as enhancers during G1ER differentiation (Figs. 4,5). Therefore, the lack of enrichment of CTCF motif is likely due to the inclusion of both hub and non-hub enhancers in promoter-centric capture experiments (Fig. 5e,f).*

*It is important to note that both CTCF/cohesin and erythroid master TFs (GATA1 and TAL1) are highly enriched in hub enhancers relative to non-hub enhancers or non-hierarchical SEs, whereas no significant difference in H3K4me1 and H3K27ac was observed (Fig. 3e). Similarly, we found that erythroid master TFs are also significantly enriched in promoter-interacting enhancers based on promoter-centric capture in G1ER cells (Figs. 5g,h). To determine the enrichment of CTCF at these promoters and enhancers, we performed CTCF ChIP- seq experiments in undifferentiated (0h) and differentiated (24h) G1ER cells. We also noted significant enrichment of CTCF occupancy in the proximity of the captured activated and repressed promoters, as well as the activated promoter-associated enhancers (Fig. S10c). These studies provide additional evidence that both CTCF/cohesin*

*and lineage master TFs are involved in E-P loops in K562 and G1ER cells. We have discussed these results in the revised manuscript (page 9, lines 1-14; page 12, lines 20-24).*

2.	Page 10, line 46; Page 11, line 39: Please supply more information (for example, number of enhancers) and reference or description for 'annotated enhancers' and indicate in figures by name or location w/respect to TSS for examples used.

*We provided detailed information about the position and number of annotated enhancers in the revised text and methods (page 10, line 32; page 25, lines 21-22). We indicated the location of each annotated enhancer with respect to TSS (+1) at Mafk and Gata2 loci in Figs. 4e,f, as suggested. We also included the detailed location (chromosome coordinates) about the captured promoters and their interacting enhancers in Table S7.*

3.	Page 11, line 42: In figure 5d are E-P interactions really more significantly and positively correlated with expression changes than 'other interactions'? It is difficult for this reader to distinguish the orange and red lines in figure 5a-d—a different color choice would help.

*We changed the color scheme in Fig. 5c,d for better illustration as suggested. To quantitatively assess the correlation between E-P interactions and RNA-seq gene expression, we performed Pearson correlation coefficient analyses. At the captured activated promoters, we observed that E-P interactions and expression were significantly and positively correlated (Pearson correlation coefficient R = 0.890, P = 0.022 by the Student’s t-test; Fig. 5c).*

*Meanwhile, the other interactions showed no significant correlations with gene expression (R = 0.698, P = 0.095).*

*Similarly, at the captured repressed promoters, we observed that E-P interactions and expression were significantly and positively correlated (Pearson correlation coefficient R = 0.905, P = 0.018; Fig. 5d). Meanwhile, the other interactions also showed positive correlations with gene expression (R = 0.871, P = 0.028), but the significance score was lower than the E-P interactions. Overall, these findings suggest that E-P interactions are more significantly correlated with gene expression changes relative to other interactions. We have provided the statistical analyses in the figure legends and results of the revised manuscript (page 11, lines 28-29; page 29, lines 17-19 and 21-24).*

4.	Figure 2a: sgRNAs are said to be close to but not overlapping enhancers but in Figure 2a it looks like the sgRNA on the right in within the enhancer.

*We apologize for the confusion and have revised the statement in the results and methods (page 7, line 6; page 20, lines 21-22). Specifically, sgRNAs were designed to be not overlapping with ‘enhancer-associated ATAC- seq peaks’. The putative enhancers were annotated based on the presence of ATAC-seq and H3K27ac ChIP-seq peaks. Since H3K27ac ChIP-seq peaks are typically broader than ATAC-seq peaks, we used ATAC-seq peak regions as the reference for putative nucleosome-free regions or TF binding sites within enhancers. The primary rationale for this design was to minimize the interference with the endogenous transcriptional machinery due to dCas9/sgRNA binding.*

Reviewer #2: In this manuscript, Liu et al aim to interrogate chromatin organization at cis-regulatory elements (CRE) such as enhancers and promoters at high resolution in a multiplexed manner. To this aim, they utilize an improved version of their previously published dCas9-based CAPTURE method, which utilizes biotinylation of dCas9 at genomic elements followed by streptavidin-based affinity purification and analysis of long-range interactions by 3C based technologies. In this paper, they improve on CAPTURE (CAPTURE 2.0) by incorporating a 75 bp biotinylation targeting sequence (BioTAP) to dCas9 that can be recognized by endogenous biotin-protein ligases (BPLs) in eukaryotic cells, which demonstrates ease of cell line engineering and increase in capture efficiency. Using the improved CAPTURE, they explore multiplexing many guide RNAs to analyze multiple CREs in a single experiment. They demonstrate that they can dissect interactions from each enhancer at the beta globin locus control region in high resolution and identify differences in organization. They further assay individual constituent enhancers at many enhancer clusters i.e. super enhancers (SE), and were able to identify qualitative and quantitative differences in long-range interactions of each CREs. They further assay promoter centered dynamic interactions in a differentiation setting. Through these demonstrations, they make a point of potential of CAPTURE in understanding chromatin organization in regulatory elements in high-resolution without any need of a priori knowledge of protein factors at said elements.

Previously, the same group have developed the CAPTURE-3C-Seq method to analyze long -range interactions from individual regulatory elements. Improving up on their previous method, here they demonstrate the potential of multiplexed CAPTURE on identifying principles of chromatin organization at important regulatory elements. This improved and multiplexed method would be of great interest to the field of chromatin organization and gene regulation.

*We appreciate the positive feedback on our manuscript.*

Comments:

1.	In the paper, CAPTURE-3C-Seq interactions are compared to Hi-C and ChIA-PET interactions. How does CAPTURE compare to recent methods such as Plac-Seq / HiChIP in capturing enhancer/ promoter centric interactions? For the browser views for the enhancer CAPTURE experiments, it would be beneficial to see published H3K27ac ChIA-PET / Plac-Seq / HiChIP interactions. As they also demonstrate in Figure 3, CTCF and cohesin is enriched in a subset of enhancers called hub enhancers whereas most of these enhancers still engage in long-range interactions.

*We appreciate these thoughtful comments, and have included additional analyses of the comparisons between CAPTURE-3C and recently published H3K27ac HiChIP data in K562 cells 1 as suggested. Specifically, we performed similar global analysis as other datasets for the % of unique PETs and on-target enrichment (Fig. S4a). Comparing with H3K27ac HiChIP, CAPTURE-3C-seq displayed higher % of unique PETs but comparable or slightly higher on-target enrichment (Fig. S4a). We also incorporated H3K27ac HiChIP data in the browser view together with all other datasets in Fig. S3. Significant long-range DNA interactions were noted at the β-globin LCR (HS1 to HS4) regions and the active β-globin genes (HBE1, HBG1 and HBG2), consistent with the enrichment of H3K27ac at these regions (Fig. S3). No public H3K27ac Plac-seq data is available for G1ER or K562 cells, and no H3K27ac HiChIP-seq data is available for G1ER cells.*

*We also provided additional clarifications on CTCF/cohesin enrichment, hub enhancers and long-range DNA interactions (page 9, lines 8-14). Specifically, while we expect that most of the enhancers engage in long-range interactions with their gene targets and/or other cis-regulatory elements, we noted that a subset of enhancers (named as hub enhancers) are associated with significantly higher frequency of long-range interactions comparing to other enhancers within a SE cluster. We adapted the H-score computational metric and systematically identified the hub enhancers and associated SEs (Fig. 3a,b). Moreover, we observed that hub enhancers are associated with stronger enrichment of CTCF/cohesin and lineage TFs (GATA1 and TAL1) compared to non-hub enhancers (Fig. 3e). Hence, our findings suggest that a subset of SE constituent enhancers (hub enhancers) are more enriched with binding of CTCF/cohesin and lineage TFs, and associated with higher frequency of long-range interactions. However, our results do not imply that hub enhancers are the only enhancers associated with CTCF/cohesin and/or long-range interactions.*

2.	Are the same gRNAs used for the multiplexed vs individual CAPTURE-3C-Seq experiments of the globin locus?

*Yes, the same sgRNAs were used for individual and multiplexed CAPTURE experiments of the β-globin LCR. The sgRNA sequences are provided in Table S1.*

3.	How many of the selected SEs / constituent enhancers are intergenic / intragenic?

*We provided information on the genomic distribution of SEs and constituent enhancers in Fig. 2a. We noted that 31%, 28% and 45% of SEs are located in the intragenic, intergenic, or both regions, respectively. Moreover, 48%, 44% and 8% of SE constituent enhancers are located in the intragenic, intergenic, or both regions, respectively (Fig. 2a). The increased portion of SEs in both intragenic and intergenic regions is likely due to the increased sizes of SEs relative to their constituent enhancers.*

4.	Do you see any other differences between hub-enhancer containing vs non-hierarchical super enhancers? Any differences in their distance to the target genes? Differences in motifs for hub vs non-hub enhancers?

*We provided more information on the comparisons between hierarchical and non-hierarchical SEs, including motif enrichment analysis and the distance to the target genes (Fig. S6c,d; page 9 lines 14-19), as suggested. Specifically, similar TF motifs were found to be enriched at hierarchical and non-hierarchical SEs (Fig. S6c), likely due to the large sizes of both hierarchical and non-hierarchical SEs containing many constituent enhancers.*

*Interestingly, we found that the distances between hierarchical SEs and their associated gene targets (mean 139kb and median 128kb) were significantly greater than the distances between non-hierarchical SEs and gene targets (mean 114kb and median 111kb) (P = 1.95E-5 by Student’s t-test; Fig. S6d; see Methods). These results suggest that the hub enhancer-containing hierarchical SEs tend to locate more distal to their gene targets, and the functional relevance of these findings in SE-mediated gene regulation warrants further investigation in future studies.*

5.	Please explain GATA1 transgene expression dynamics upon estradiol treatment in Gata1-null G1ER cells. Prior to differentiation, at time 0 of estradiol treatment, how much Gata1 is expressed that you see enrichment at GATA1-ChIP Seq?

*We appreciate this thoughtful comment, and have provided more information on GATA1 expression kinetics and chromatin occupancy in G1E and G1ER cells in the revised manuscript (Fig. S7a,b). Specifically, as previously described 2-4 G1ER is a G1E subclone that constitutively expresses an estradiol-activated form of GATA1 fused to the estrogen receptor ligand binding domain (GATA1-ER). Upon treatment with β-estradiol, GATA1-ER transgene is activated to induce erythroid differentiation of G1ER-ER4 cells 2-4. The expression of GATA1-ER protein was previously noted to be expressed at comparable levels in undifferentiated (0h) and differentiated (β-estradiol treatment for 24h) G1ER-ER4 cells, and absent in G1E cells (see Fig. 1B in reference #4) 4. Consistent with these findings, we observed that the mRNA for GATA1-ER transgene is expressed at comparable levels in undifferentiated (0h) and differentiated (24h) G1ER-ER4 cells but absent in G1E cells (Fig. S7a).*

*To determine how GATA1’s expression correlates with chromatin occupancy in G1ER-ER4 cells, we re- analyzed the published GATA1 ChIP-seq data 5-7 in G1E, undifferentiated (0h) and differentiated (24h) G1ER-ER4 cells (Fig. S7b). Comparing with input DNA, there are very few GATA1 binding peaks in G1E cells consistent with the absent GATA1 expression. In contrast, comparable numbers of GATA1 ChIP-seq peaks were observed in undifferentiated (0h) and differentiated (24h) G1ER-ER4 cells (Fig. S7b, left). By overlap analysis, we observed that 48.0% of GATA1 ChIP-seq peaks are shared between undifferentiated (0h) and differentiated (24h) G1ER- ER4 cells, whereas the other peaks are unique to undifferentiated or differentiated cells, respectively (Fig. S7b, right). These results are consistent with previous analyses 6, suggesting that GATA1 activates and represses different set of gene targets at different stages of erythroid differentiation of G1ER-ER4 cells. We have included these results in the revised manuscript (page 9, lines 26-34).*

*Reference:*

*1.	Mumbach, M.R. et al. Enhancer connectome in primary human cells identifies target genes of disease-associated DNA elements. Nature Genetics 49, 1602-1612 (2017).*

*2.	Tsang, A.P. et al. FOG, a multitype zinc finger protein, acts as a cofactor for transcription factor GATA-1 in erythroid and megakaryocytic differentiation. Cell 90, 109-119 (1997).*

*3.	Gregory, T. et al. GATA-1 and erythropoietin cooperate to promote erythroid cell survival by regulating bcl-xL expression.*

*Blood 94, 87-96 (1999).*

*4.	Welch, J.J. et al. Global regulation of erythroid gene expression by transcription factor GATA-1. Blood 104, 3136-3147 (2004).*

*5.	Cheng, Y. et al. Erythroid GATA1 function revealed by genome-wide analysis of transcription factor occupancy, histone modifications, and mRNA expression. Genome Research 19, 2172-2184 (2009).*

*6.	Jain, D. et al. Dynamics of GATA1 binding and expression response in a GATA1-induced erythroid differentiation system.*

*Genomics Data 4, 1-7 (2015).*

*7.	Wu, W. et al. Dynamics of the epigenetic landscape during erythroid differentiation after GATA1 restoration. Genome Research 21, 1659-1671 (2011).*

**Second round of review**

**Reviewer 1**

Authors have responded thoroughly to reviewers comments and provided appropriate revisions to the manuscript.

NB: According to text on page 11, figure S10 should have another panel. Present panel C appears to represent S10D and S10C, about CTCF is missing.

**Reviewer 2**

In this revised manuscript, Liu et al interrogated chromatin organization at cis-regulatory elements (CRE) at high resolution in a multiplexed manner. By utilizing biotinylation of dCas9 at genomic elements with multiple guide RNAs, they were able to capture long-range interactions by 3C based technologies in a multiplexed manner in a single experiment. They not only demonstrate interactions from each enhancer of the beta-globin locus, but also explore individual constituent enhancers at many enhancer clusters i.e. super enhancers (SE), to identify qualitative and quantitative differences in long-range interactions of each CREs. They further assay promoter centered dynamic interactions in a differentiation setting. Through these demonstrations, they make a point of potential of CAPTURE in understanding chromatin organization in regulatory elements in high-resolution without any need of a priori knowledge of protein factors at said elements.

The findings of the manuscript, and the improved CAPTURE method to identify interactions from multiplex loci are of general interest to the gene regulation and chromatin organization field. From the first manuscript, the authors have addressed all the issues and questions present in the reviewers' comments and through this process strengthened their work.

Minor comment:

Page 11, lines 11-13 "Furthermore, refined kinetic analyses demonstrate that changes in gene expression occur in close cooperation with gained/lost enhancer-promoter interactions and precede significant changes in epigenetic landscapes or chromatin accessibility" Could you specify whether this is only for the loci shown, and if so does it imply the promoters or the interacting enhancers? It seems at Figure 5i,j at global levels, you already show significant changes in chromatin accessibility and H3K27ac levels at the activated promoters and their interacting enhancers as early as 2 hours.

**Authors Response**

**Point-by-point responses to the reviewers’ comments:**

Comment

*Response*

**Reviewer 1**

Authors have responded thoroughly to reviewers comments and provided appropriate revisions to the manuscript.

NB: According to text on page 11, figure S10 should have another panel. Present panel C appears to represent S10D and S10C, about CTCF is missing.

*1) With regard to reviewer 1’s minor comment (page 11, line 19), we specified that the statement is only for the loci shown by adding “at several independent loci” to the statement.*

**Reviewer 2**

In this revised manuscript, Liu et al interrogated chromatin organization at cis-regulatory elements (CRE) at high resolution in a multiplexed manner. By utilizing biotinylation of dCas9 at genomic elements with multiple guide RNAs, they were able to capture long-range interactions by 3C based technologies in a multiplexed manner in a single experiment. They not only demonstrate interactions from each enhancer of the beta-globin locus, but also explore individual constituent enhancers at many enhancer clusters i.e. super enhancers (SE), to identify qualitative and quantitative differences in long-range interactions of each CREs. They further assay promoter centered dynamic interactions in a differentiation setting. Through these demonstrations, they make a point of potential of CAPTURE in understanding chromatin organization in regulatory elements in high-resolution without any need of a priori knowledge of protein factors at said elements.

The findings of the manuscript, and the improved CAPTURE method to identify interactions from multiplex loci are of general interest to the gene regulation and chromatin organization field. From the first manuscript, the authors have addressed all the issues and questions present in the reviewers' comments and through this process strengthened their work.

Minor comment:

Page 11, lines 11-13 "Furthermore, refined kinetic analyses demonstrate that changes in gene expression occur in close cooperation with gained/lost enhancer-promoter interactions and precede significant changes in epigenetic landscapes or chromatin accessibility" Could you specify whether this is only for the loci shown, and if so does it imply the promoters or the interacting enhancers? It seems at Figure 5i,j at global levels, you already show significant changes in chromatin accessibility and H3K27ac levels at the activated promoters and their interacting enhancers as early as 2 hours.

*2) With regard to reviewer 2’s minor comment, we double checked to make sure that the figure panels in Fig. S10 are appropriately organized and cited in the text.*
